# Enhanced access to the human phosphoproteome with genetically encoded phosphothreonine

**DOI:** 10.1038/s41467-022-34980-5

**Published:** 2022-11-24

**Authors:** Jack M. Moen, Kyle Mohler, Svetlana Rogulina, Xiaojian Shi, Hongying Shen, Jesse Rinehart

**Affiliations:** 1 Department of Cellular & Molecular Physiology, Yale School of Medicine, New Haven, CT 06520 USA; 2 Systems Biology Institute, Yale University, New Haven, CT 06516 USA; 3 Wu Tsai Institute, Yale University, New Haven, CT 06520 USA

**Keywords:** Synthetic biology, Protein-protein interaction networks, Protein-protein interaction networks

## Abstract

Protein phosphorylation is a ubiquitous post-translational modification used to regulate cellular processes and proteome architecture by modulating protein-protein interactions. The identification of phosphorylation events through proteomic surveillance has dramatically outpaced our capacity for functional assignment using traditional strategies, which often require knowledge of the upstream kinase a priori. The development of phospho-amino-acid-specific orthogonal translation systems, evolutionarily divergent aminoacyl-tRNA synthetase and tRNA pairs that enable co-translational insertion of a phospho-amino acids, has rapidly improved our ability to assess the physiological function of phosphorylation by providing kinase-independent methods of phosphoprotein production. Despite this utility, broad deployment has been hindered by technical limitations and an inability to reconstruct complex phopho-regulatory networks. Here, we address these challenges by optimizing genetically encoded phosphothreonine translation to characterize phospho-dependent kinase activation mechanisms and, subsequently, develop a multi-level protein interaction platform to directly assess the overlap of kinase and phospho-binding protein substrate networks with phosphosite-level resolution.

## Abstract (web summary)

Protein phosphorylation is a ubiquitous post-translational modification used to regulate cellular processes and proteome architecture by modulating protein-protein interactions. Here the authors optimize genetically encoded phosphothreonine to study the regulation of CHK2 kinase using large-scale DNA arrays that enable phosphoproteome expression techniques to identify sitespecific overlap between CHK2 substrates and 14-3-3 interactions.

## Introduction

Regulation of cellular signaling cascades occurs through complex protein interactions facilitated by an array of post-translation modifications (PTMs). Protein phosphorylation, in particular, is central to PTM-mediated regulation of signaling cascades and associated protein-protein interactions (PPIs)^1,2^. Advances in proteomics have increased our ability to identify phosphorylation sites (phosphosites) that participate in signaling, yielding an expansive map of the human phosphoproteome. However, the identification of these sites has dramatically outpaced our ability to connect them to underlying interactions. Protein kinases (which catalyze the ATP-dependent addition of phosphate to an amino acid), and conversely, protein phosphatases (which catalyze hydrolysis-dependent removal of phosphate from an amino acid) enact phospho-regulatory mechanisms to mediate intracellular function^3,4^. Connecting these essential mechanisms to the proteome has been challenging due to a lack of comprehensive information regarding the identity of upstream activating kinases, the inherently dynamic expression and PTM state of kinase and phosphatase effectors, and the modulation of PPIs by non-catalytic phospho-binding proteins^5–7^. These regulatory complexities underscore the need for technological advances that enable the rapid investigation of interconnected phospho-mediated physiology on a global scale.

Although chemical synthesis of full-length phosphoproteins is limited, phosphopeptide arrays have been successfully deployed for mechanistic insight but have remained cost-prohibitive for large-scale investigations. Orthogonal translation systems (OTS) provide a rapid, cost-efficient, renewable solution to this problem^8^. The first of these systems utilized phosphoseryl-tRNA synthetase (pSerRS) from *Methanococcus maripaludis* to aminoacylate pSer onto a modified amber (UAG) suppressor tRNA^Cys^ from *Methanococcus jannaschii*^9^. These early systems suffered from low fidelity and poor efficiency due to problems with substrate specificity and incomplete orthogonality. The first solution to these problems came through the evolution of pSerRS and tRNA^pSer^ for enhanced fidelity^10^. The second advancement was the development of a genomically recoded *Escherichia* *coli* (C321.ΔA) where all 321 instances of the TAG codon were recoded to TAA, enabling deletion of the UAG-terminating release factor 1 (RF1 or ΔA), thereby decreasing competition for pSer incorporation at UAG codons^11^. Previous work from our lab has expanded the applications of the pSerOTS to enable the large-scale production of a recombinant phosphopeptide library containing all known human pSer sites as 31 amino acid peptides^8^. This technology enabled the development of a technique termed Hi-P, in which the pSer peptide library is used in a bimolecular fluorescence complementation assay with a phospho-binding domain to rapidly identify phosphorylation-dependent protein-protein interactions. Beyond pSer, the development of OTSs for incorporating phosphotyrosine (pTyr) has proven particularly challenging^12–14^. Iterations of pTyrOTSs utilize an *M. jannaschii* TyrRS and tRNA^Tyr^ pair, relying on either phosphatase deletion strains^12^ or dipeptide mediated transport^14^ to drive intracellular pTyr. However, it is possible to use pTyr mimetics, e.g., p-carboxymethyl-l-phenylalanine (CMF)^15^, as they cross the *E. coli* periplasmic space without modification, but they are often poor substitutes for pTyr^16,17^. Despite concerted effort to develop more phospho-amino acid OTSs over past decade, only one phosphothreonine OTS (pThrOTS) has been developed^18^. However, this pThrOTS has only been validated with a small set of phosphoproteins and has only been tested in *E. coli* B strain lineages. Further expansion of pThrOTSs is required to enhance access to pThr-dependent processes within the cell and, more broadly, deepen our understanding of phosphorylation-mediated cellular physiology.

Using a previously characterized phosphothreonyl aminoacyl-tRNA synthetase (pThrRS) and tRNA^pThr^ pair^18^, we developed a rationally designed and optimized OTS capable of facilitating high levels of pThr incorporation. Together with several strain innovations that maximized the production of phosphoproteins, we were able to perform pThr-mediated PPI studies and characterize mechanisms of activation for checkpoint kinase 2 (CHK2). Coupling our ability to produce active kinases with our platform to identify phosphorylation-dependent PPIs, we developed Hi-P+ and profiled the overlap between CHK2 substrate phosphorylation and 14-3-3β phosphorylation-dependent interactions. Overall, our work demonstrates the utility of application-driven phospho-amino acid-based OTS development and highlights how genetic programming of the human phosphoproteome allows for the simultaneous, multi-level characterization of convergent kinase and phospho-binding protein interactions with phosphosite resolution.

## Results

### pThrOTS development and strain optimization for enhanced pThr incorporation

Leveraging advances from prior pSerOTS work^19–21^, we aimed to incorporate components from the existing pThrOTS^18^ into a unique OTS architecture to ultimately enhance access to pThr-mediated biology. To establish baseline functionality of the initial pThrOTS components, we first incorporated them into our previously developed OTS framework consisting of the pThrRS and pSer elongation factor 1 (EF-pSer1) under control of a high-level mutant constitutive TRC promoter (TRC*)^9,18,21^. EF-pSer1, along with a further optimized variant (EF-pSer21), were evolved from a bacterial elongation factor (EF-Tu) to facilitate efficient delivery of bulky, negatively charged phospho-aminoacyl-tRNA to the ribosome^9,10^. The pThrOTS system relies upon the bacterial enzyme pduX to catalyze the ATP-dependent conversion of free L-threonine to pThr^18^, creating stable intracellular pools of pThr substrate. Our initial pThrOTS framework placed pduX under the control of the arabinose inducible promoter (pBAD). A constitutive lpp promoter controlled the expression of tRNA. We conducted our preliminary pThrOTS optimizations in a C321.ΔA strain with a serC deletion (C321ΔserC), the enzyme which converts metabolic precursors to pSer during Ser biosynthesis^22^. Deleting serC was previously shown to be essential for decreasing intracellular levels of pSer, thereby decreasing competition with pThr in the pThrRS active site^18^. Using this framework, we systematically modified OTS vector components to characterize their impact on phosphoprotein production. Modifications included: (1) placing the pThrRS:EF-pSer operon under the control of the low-level constitutive promoter glnS*; (2) replacing EF-pSer1 with EF-pSer21; (3) substituting tetracycline resistance for kanamycin resistance; (4) placing pduX expression under the control of the high-level constitutive promoter OXB20; (5) insulating the tRNA cassette with separate T1 and T7 terminators.

To assess pThr incorporation facilitated by a subset of OTS variants, we utilized our Mass Spectrometry Reporter for Exact Amino acid Decoding (MS-READ) reporter protein^23^. The reporter is a 6x His tagged, TAG-containing elastin-like polypeptide, green fluorescent protein fusion that can be used to monitor relative pThr-protein expression by immunoblot and quantify amino acid incorporation at UAG codons by MS. To quantitatively assess OTS function, we expressed the reporter protein with each OTS variant and analyzed pThr incorporation relative to other amino acids incorporated at the UAG codon via MS (Supplementary Fig 1A). We observed the highest levels of pThr incorporation when pThrRS was under control of the TRC*.

**Fig. 1:**
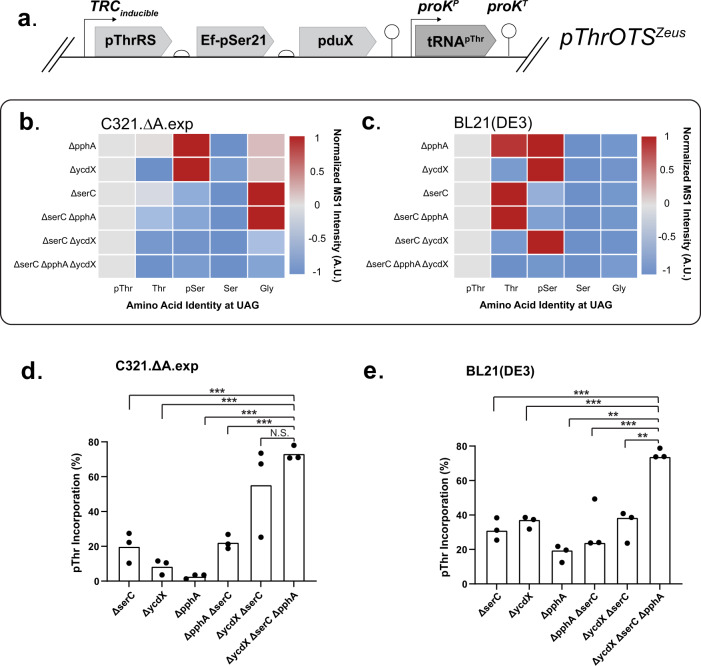
Development and characterization of a pThrOTS-strain combination. **a** Schematic representation of pThrOTS^Zeus^ polycistronic operon and tRNA cassette. Analysis of MS-READ reporter phosphorylation and expression for **b** C321.ΔA.exp and **c** BL21 (DE3). Values are scaled to pThr intensity within strains (*N* = 3). Percent pThr incorporation (*N* = 3) for knockout strains expressing pThrOTS^Zeus^ and MS-READ reporter in either **d** C321.ΔA.exp or **e** BL21(DE3). Significance determined by two-tailed student’s *T*-test (equal variance) noted by *p < 0.05, **p < 0.01, ***p < 0.001 or non-significant (NS) *P* values from left (∆serC) to right (∆ycdX ∆serC) for (**d**): 6.75E−04, 4.76E−05, 9.11E−06, 1.1E−04 and **e** 4.34E−04, 1.16E−04, 6.08E-05, 7.47E−03, 1.88E−03.

Because promoter activity emerged as a mediator of pThr incorporation, we wanted to assess the impact of promoter strength variation on pThr production. We constructed pduX expression vectors with diverse transcriptional promoters and measured the levels of pThr metabolite production by LC-MS/MS (Supplementary Fig 1B). Three promoters were chosen based on diverse activities; (1) pBAD, a moderately high inducible promoter that could function independently of pThrRS and EF-pSer expression in an OTS, (2) OXB20, a constitutively high promoter used in a previous pThrOTS^18^, (3) TRC, an inducible high expression promoter used to drive pSerRS and EF-pSer in previous OTS designs^21^. When inducible promoters were maximally induced, we observed that the TRC promoter (Supplementary Fig 1B, P4) consistently facilitated the highest levels of pThr production (Supplementary Fig 1B). As TRC drove the highest levels of intracellular pThr production and had already been implemented to regulate expression of pThrRS and EF-pSer, we sought to combine the architecture into a single TRC-inducible operon, thereby minimizing extraneous genetic elements. The optimized variant (pThrOTS^Zeus^) placed *pduX* in a polycistronic operon with *pThrRS* and *EF-pSer21* under transcriptional control of the TRC promoter (Fig. 1a). We also utilized the native *E. coli* proK tRNA promoter and terminator pair to enhance tRNA production in OTSs^24^.

To identify putative host factors that could compromise pThr protein production, we screened OTS performance in 16 strains containing targeted deletions for candidate genes with database annotations for phosphatase function by Phos-tag-SDS-PAGE (which separates proteins based on phosphorylation status) followed by immunoblot detection of the 6x His tag. Among the 16 individual phosphatase deletion strains, we saw minimal, to no change in the ratio of phosphorylated to non-phosphorylated recombinant reporter protein (Supplementary Fig 2). Due to these results and technical limitations of this screening approach, we shifted towards a focused analysis of enzymes known to improve pThrOTS performance in other contexts. We chose to delete *serC* because of its canonical role in producing high levels of pSer^22^, the major competing substrate for pThrRS; *ycdX* due to its free pThr phosphatase activity^18^; and *pphA* due to its homology with the highly promiscuous lambda phosphatase^25^.

Focusing on the three target genes, we next measured the impact of these candidates on steady-state pThr-protein levels by individual, or combinatorial, deletion in the BL21(DE3) and C321 strain (C321.ΔA.exp) backgrounds (Supplementary Fig 3). To assess phosphoprotein production, we used MS-READ to provide quantitative information on pThr decoding fidelity and native amino acid misincorporation at UAG codons in each deletion strain background. From these data, we constructed heat maps displaying precursor ion intensity as a proxy for amino acid incorporation frequency to identify gene deletions linked to specific amino acid incorporation events at UAG codons (Fig. 1b, c). Incorporation values within the heatmap are scaled to the relative abundance of pThr for the same peptide, the data is also represented as %pThr incorporation relative to all amino acids identified (Fig. 1d, e). The triple knockouts facilitated the highest consistent pThr-protein production in both C321 and BL21, however, the double knockout, ∆ycdX ∆serC, enhanced pThr incorporation only in the C321 background. This can be explained by the enrichment of pSer observed in the ∆ycdX ∆serC BL21 background that compromises pThr purity (Fig. 1c). We found that many of the C321 deletion strains showed elevated levels of Gly misincorporation that were not present in BL21 (Fig. 1b, c). To understand the relative rates misincorporation events, we deployed vectors containing only the tRNA cassette, i.e., absent pThrRS, EF-pSer, and pduX, in each TKO strain with our MS-READ reporter. C321 exhibits higher rates of Gly and Gln misincorporation, while conversely, BL21 displayed considerably lower levels of Gly and Gln misincorporation at UAG codons, presumably due to the competition by release factor 1, a component absent in C321 strains (Supplementary Fig 4). To understand how these variables may impact protein yield, we performed quantitative assessments of pThrOTS^Zeus^ protein production in BL21^TKO^ and C321^TKO^. The BL21^TKO^ strain produced more recombinant protein than the C321^TKO^ strain, yielding ~90 and 58 mg/L, respectively (Supplementary Fig 5). Using Phos-tag SDS-PAGE gels and immunoblot, we calculated the approximate portion of recombinant phosphoprotein to be ~37 and 20 mg/L in BL21^TKO^ and C321^TKO^, respectively. This work demonstrates the robustness of our OTS-strain combination. Based on these findings, we chose to take pThrOTS^Zeus^ and the TKO strains forward for further applications.

### Large-scale expression of a recombinant human pThr peptide library

In prior work, we developed a pSer containing peptide library encoding ~110,000 unique phosphosites identified across the human phosphoproteome^8^. This technology enabled the rapid profiling of phospho-binding domain substrates^8^ via genetically encoded pSer and kinase substrates via genetically encoded Ser^26^. To expand the functional scope of this technology, we sought to create a similar system for pThr. To this end, we extracted pThr phosphosite data from the PhosphoSitePlus database to guide construction of a codon-optimized phosphosite library DNA array corresponding to 57,536 unique pThr sites and flanking 15aa sequences (resulting in 31aa for each phosphosite)^8,27^. Due to the absence of well-characterized Thr amber suppressor tRNAs, a duplicate library was synthesized by substituting the central TAG codon for ACC to enable expression of a non-phosphorylated, Thr-containing phosphosite library.

We previously developed the high-throughput interactome of phosphoproteins (Hi-P) method for screening individual pSer phosphosites with a single phospho-binding domain (PBD)^8^. This system utilized a bimolecular fluorescence complementation framework in which the PBD (fused to the C-terminal half of a split-mCherry) interacts with a phosphosite (fused to the N-terminal half of a split-mCherry) to reconstitute the fluorescent mCherry protein, enabling enrichment of productive interactions via fluorescence-activated cell sorting (FACS). Because Hi-P enables high-throughput testing of phosphoprotein interactions, we deployed it as an initial assessment for the ability of each TKO background strain to facilitate phosphothreonine-dependent protein interactions (Supplementary Fig 6A–C). Using a well validated Hi-P PBD (14-3-3β)^8^, we were able to rapidly compare the performance of pThrOTS^Zeus^ in both BL21^TKO^ and C321^TKO^ strains. We found that neither strain was able to reproducibly enrich phospho-dependent PPIs (Supplementary Fig 6B, C). The BL21^TKO^ strain failed to produce a shift in fluorescence intensity from the starting population, while the C321^TKO^ strain failed to maintain a fluorescence intensity following the first round of enrichment. Recent work on the optimization of a pSerOTS system^20^ indicated that the stringent response was active in many OTS-containing strains. Activation of the stringent response was a significant concern since it inhibits protein synthesis potentially hindering recombinant phosphoprotein production^28^. We hypothesized that this stress response mechanism might also be a factor because of the increased translational demand of the OTS and Hi-P components. To investigate this hypothesis, we knocked out the primary tRNA-mediated stringent response effector, *relA*^29^, in both C321.ΔA.exp^TKO^ and BL21^TKO^ strains, generating quadruple knockout (QKO) strains of C321^QKO^ and BL21^QKO^. We then repeated our Hi-P experiment with the QKO strains, which resulted in a consistent, productive shift in population fluorescence intensity following enrichment for C321^QKO^ indicating that the *relA* knockout restored Hi-P function (Supplementary Fig 6D). Knocking out *relA* in BL21 failed to restore Hi-P function (Supplementary Fig 6E). Absence of Hi-P function in all of the BL21 backgrounds tested suggests that the recoded genome in the C321^QKO^ strain is an additional advantage for Hi-P. Altogether, the Hi-P performance and proteomics data indicated that the *relA* knockout in C321^QKO^ background is required for productive pThr-mediated Hi-P.

After identifying strain modifications that enhance pThr phosphosite library production, we next sought to establish the practical depth and complexity of our phosphosite libraries. We first expressed and characterized the analogous non-phosphorylated Thr phosphosite library. We identified 25,378 Thr-phosphosites or ~44% of the theoretical library at the protein level. Following characterization of our Thr library, we used phosphopeptide-enrichment strategies coupled to MS analysis to profile the expression of the pThr library. We initially identified a limited number of phosphosites (~3200). Although we had found low-level Gly and Gln incorporation in C321 strains (Fig. 1), we could only identify 58 misincorporated Gly-containing peptides and 40 misincorporated Gln-containing peptides within our pThr library datasets. Thus, it did not appear that misincorporation events were limiting the diversity of phosphopeptide expression. Since it is well known that phosphorylation can decrease ionization efficiency^28^, we theorized that this might be an issue limiting phosphosite detection.

**Fig. 2:**
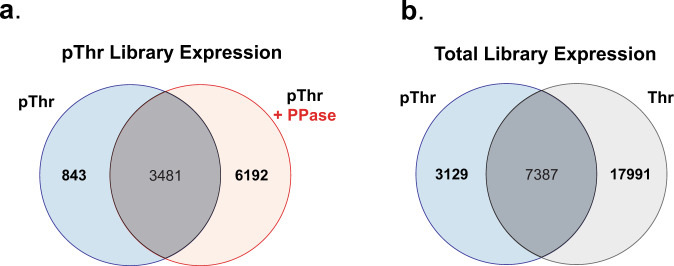
Development and expression of a human pThr phosphosite library. **a** Venn diagram comparing pThr phosphosites identified with and without phosphatase treatment. Data were combined from two biological replicates, each divided between phosphatase treatment groups. **b** Combined coverage of phosphosites identified between all pThr and Thr datasets. Data were combined from two biological replicates for each library type with Thr data encompassing six high pH reversed-phase fractions each and the pThr data as in part A.

In order to test the effects of pThr-mediated ion suppression, we relied upon an approach involving enzymatic dephosphorylation of phosphopeptides with Lambda phosphatase following phospho-enrichment^28^. Lambda phosphatase treatment resulted in the identification of an additional ~5000 phosphosites, supporting the hypothesis that phosphorylation is a complicating factor during peptide ionization. To better understand the effect of phosphatase treatment on peptide ionization efficiency, we utilized a high-throughput peptide analyzer^30^ to calculate a diverse array of physicochemical values for each tryptic fragment in silico. The phosphatase treatment enabled the identification of peptides of a greater length and mass (Supplementary Fig 7A). When comparing the isoelectric point (pI) to charge density for experimentally identified peptides, we noticed an increase in abundance of peptides with low pIs and low charge density following phosphatase treatment (Supplementary Fig 7B). After generating a comprehensive database of physicochemical properties for all theoretical tryptic peptides within the Thr library, we cross-referenced these properties with those from experimentally determined tryptic peptides across both libraries, e.g., Thr and pThr, to identify peptides with unique physicochemical properties missing between theoretical and experimental datasets. We found that the phosphatase treatment dramatically enhanced identification of peptides with a non-phosphorylated pI range of 4–5.5, i.e., made the pThr dataset more similar to Thr (shift from blue to red compared to gray in Supplementary Fig 7C). Interestingly, when the aggregate pThr and Thr data were plotted together (Supplementary Fig 8A), we found that the pThr dataset was missing phosphosite peptides with a pI of ~8. Using a motif analysis^31^, we identified the aggregate phosphosites as having a strong Cys motif around the central pThr residue (Supplementary Fig 8B). We theorized that during process of sample preparation for MS analysis, phosphorylation might inhibit the capping of Cys residues prior to tryptic peptide generation. Indeed, re-searching the dataset with a variable capping modification led to identification of an additional ~2,300 peptide IDs (Supplementary Fig 8C), with ~10,500 total pThr peptide IDs (Fig. 2a, b).

Due to the differences seen in the pI following phosphatase treatment, we wanted to better understand these differences in the context of phosphorylation. We calculated the pI for the subset of tryptic peptides experimentally identified in both the Thr and pThr + phosphatase treatment datasets but absent in its phosphorylated state (Supplementary Fig 8D). Upon the inclusion of pThr, these peptides shifted to substantially lower pIs, often below 4. Although we were unable to calculate the charge density for phosphopeptides, it is not inconceivable that the negative charge imparted by the phosphate group would further reduce the ionization efficiency. One interesting observation that arose from our experimentally derived datasets was an inability to identify peptides with a pI of ~12 (Supplementary Figs 7B, 8A, 8C), despite analysis of our theoretical Thr library peptides showing that the peptides falling within this pI range should be present (Supplementary Fig 9A). Analysis of experimentally derived peptides from total *E. coli* proteomes run as quality control before and after the library analysis also contained tryptic peptides with a pI of ~12 (Supplementary Fig 9B). Unfortunately, we were unable to determine the experimental or technical limitations that resulted in the missing peptides window. None the less, we were able to identify 28,507 peptides between our Thr and pThr libraries (Fig. 2b), representing ~50% of our theoretical phosphosite library coverage. Surprisingly, a subset of peptides could only be identified with pThr present; likely the result of phosphosite-specific differences in protein abundance during OTS-mediated protein translation or changes to the physicochemical properties of the peptides in a manner that altered peptide ionization or detection. DNA sequence (Supplementary Fig 9C) and protein sequence (Supplementary Fig 9D) analysis of phosphosites missing from all experimentally derived datasets failed to identify any unique sequence determinants that would limit protein expression. As this data was collected using data-dependent acquisition methods it is difficult to say whether the differences we observed are due to the stochastic nature of peptide sampling or other factors. We believe that additional replicates, peptide fractionation approaches, and analysis strategies could further enhance the depth of MS coverage. Overall, these data illustrate the depth of our phosphosite library and highlight important technical considerations for recombinant pThr-protein analysis by MS. Moreover, this work shows how our genetically encoded phosphosite libraries could be used to further improve MS technologies.

### Development of Ala suppressor tRNA for phospho-ablative programming

**Fig. 3:**
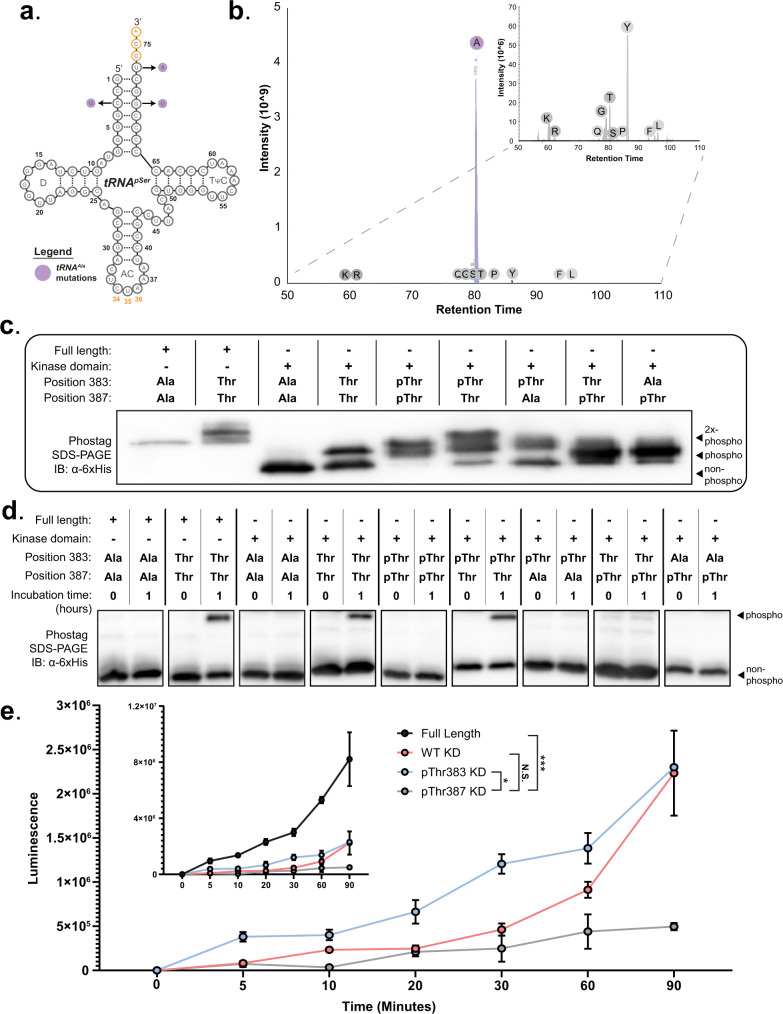
Genetically encoded phosphorylation alters CHK2 kinase activity. **a** Three substitutions (purple) in the acceptor stem of ${{{{{{\rm{tRNA}}}}}}}_{{{{{{\rm{CUA}}}}}}}^{{{{{{\rm{pSer}}}}}}}$ result in ${{{{{{\rm{tRNA}}}}}}}_{{{{{{\rm{CUA}}}}}}}^{{{{{{\rm{Ala}}}}}}}$ suppressor activity. **b** LC-MS/MS analysis and total ion chromatogram of ${{{{{{\rm{tRNA}}}}}}}_{{{{{{\rm{CUA}}}}}}}^{{{{{{\rm{Ala}}}}}}}$-mediated Ala incorporation at UAG codons within the MS-READ reporter. **c** The relative phosphorylation status of CHK2 variants separated via Phos-tag SDS-PAGE and visualized by immunoblot against 6xHis (*N* = 2). **d** Phos-tag SDS-PAGE separation of CDC25C substrate pre- and post-incubation with CHK2, visualized by immunoblot against 6xHis (*N* = 2). **e** ADP-Glo kinase assay comparing substrate phosphorylation over 90 min (*N* = 3, error bars shown as ± 1 SE). Significance determined by nonparametric one-way ANOVA with Dunn’s correction for multiple comparisons noted by *p < 0.05, **p < 0.01, ***p < 0.001 or non-significant (NS), pThr387 × pThr383 *p* value = 0.0389, pThr387 × Full-Length *p* value = 0.0006. Central callout contains full-length CHK2 luminescence for comparison to KD variants.

Previous work demonstrated that targeted mutations in tRNA^pThr^ could decrease misaminoacylation by native *E. coli* aminoacyl-tRNA synthetases, thus enhancing the fidelity of pThr incorporation^18^. In our hands, tRNA^pThr^ facilitated high levels of Gly incorporation at UAG codons (Supplementary Fig 4). In an attempt to reduce misincorporation that resulted from Gly misaminoacylation, we redesigned tRNA^pSer^ from a previously characterized pSerOTS^21^ by adding recognition elements for D-aminoacyl-tRNA deacylase (DTD), whose secondary function is to prevent Gly misincorporation by hydrolyzing mis-glycylated tRNAs (Fig. 3a)^32^. Using MS-READ, we profiled the fidelity of our modified tRNA and, surprisingly, discovered that our modified tRNA functioned as a robust Ala suppressor tRNA (Fig. 3b). Further interrogation of Alanyl-tRNA Synthetase (AlaRS) recognition elements showed that the G3:U70 pair is a major determinant for AlaRS^33^. The simple conversion ${{{{{{\rm{tRNA}}}}}}}_{{{{{{\rm{CUA}}}}}}}^{{{{{{\rm{pSer}}}}}}}$ to ${{{{{{\rm{tRNA}}}}}}}_{{{{{{\rm{CUA}}}}}}}^{{{{{{\rm{Ala}}}}}}}$ supports the theory that a wide range of tRNAs could be easily mutated to accommodate aminoacylation by AlaRS. The serendipitous development of an Ala suppressor tRNA provided a mechanism to program non-phosphorylatable residues in recombinantly expressed proteins. Thus, we reasoned that our ${{{{{{\rm{tRNA}}}}}}}_{{{{{{\rm{CUA}}}}}}}^{{{{{{\rm{Ala}}}}}}}$ and pThrOTS^Zeus^ could enable us to make either active phosphoprotein or inactive non-phosphorylatable protein using the same TAG-containing construct.

### Co-translational insertion of pThr or Ala provides insights into CHK2 kinase activation

Phosphorylation by protein kinases is integral to signal transduction and cellular processes in mammalian cells. The serine/threonine kinase, CHK2, is a prime example of phosphoregulatory mechanisms for cellular processes through its role in the modulation of cell cycle progression and DNA repair pathways^34,35^. ATM phosphorylates CHK2 Thr68 in response to DNA damage, which in turn enables CHK2 dimerization and trans-phosphorylation of activation loop residues Thr383 and Thr387^36,37^. Previous reports of recombinant CHK2 expression identified that full-length CHK2 undergoes trans-phosphorylation during protein expression and purification, yielding catalytically active kinase^38^. Trans-autoactivation at two regulatory pThr sites is difficult to dissect with traditional mutagenesis because amino acid substitutions that eliminate Thr sites can interfere with activation loop function. We aimed to use genetically encoded pThr to probe the precise mechanisms of CHK2 activation using the kinase domain (KD). Directly encoding pThr enables the precise attribution of individual phosphorylation events within the activation loop that contribute to the regulation of kinase function. We constructed a series of KD variants to dissect the contributions of each phosphorylation state to overall kinase activity (Fig. 3c). Our initial analysis recapitulated previous observations of hyperphosphorylation in full-length CHK2 and autoactivation of the KD. Double Thr383Ala and Thr387Ala substitution inactivated both the full-length kinase and the KD. Phos-tag SDS-PAGE gels of the wild-type KD (Thr383/Thr387) suggested that only a single activation loop Thr was auto-phosphorylated, and MS analysis localized the phosphorylation site to Thr383 (Supplementary Fig 10A). Direct synthesis of pThr383/Thr387 CHK2 KD appeared to result in hyperphosphorylation, while a pThr383/Ala387 mutant more closely resembled the wildtype KD shift (Fig. 3c, lane 4 compared to lanes 6&7). We expected the insertion of pThr387 into the KD to produce a phosphorylation profile similar to pThr383/Thr387 KD; however, we only observed a single phosphorylation state (Fig. 3c, columns 8&9). MS analysis confirmed that the genetically programmed pThr387 was the only phosphorylation event within the activation loop (Supplementary Fig 10A). We were unable to confirm the presence of dual pThr incorporation into the activation loop of full-length CHK2 nor pThr383/pThr387 KD by MS, presumably due to the previously discussed complexities of pThr phosphopeptide ionization. However, gel shift analysis supported dual pThr incorporation and produced a unique mixture of singly and doubly phosphorylated KD that was not recapitulated by any other variant. These unusual results suggest that position 383 may be critical to a unique and previously uncharacterized mechanism of CHK2 activation.

To assess the activity of the KD variants, we performed in vitro phosphorylation assays using the well-characterized CHK2 substrate CDC25C (Fig. 3d)^35^. Across all CHK2 variants, only full-length CHK2, wildtype KD, and pThr383 KD phosphorylated the CDC25C substrate. To examine the activity of these CHK2 variants more closely, we used an ADP-Glo assay to monitor the kinetics of CDC25C substrate phosphorylation by our active variants (full-length CHK2, wildtype KD, and pThr383 KD) and one inactive variant (pThr387 KD) (Fig. 3e). Full-length CHK2 displayed the highest level of activity, followed by pThr383 KD and then WT KD. The pThr387 KD variant failed to appreciably phosphorylate the CDC25C substrate. Our kinase assays support the hypothesis that CHK2 catalytic activity may be solely dependent on pThr383. We also observed that while pThr383/Thr387 was active, pThr383/Ala387 was inactive. Upon further investigation, we found that insertion of Ala at either position 383 or 387 ablated the KD activity (Fig. 3d & Supplementary Fig 10B). However, Ala substitution at Thr225, another well-characterized CHK2 phosphosite^39^, had no impact on KD activity or autophosphorylation. Our data indicate that Ala substitution at either activation loop position results in kinase inactivation but through a mechanism more complicated than phosphosite ablation. The collective CHK2 data show that pThr383 is necessary and sufficient to activate CHK2 and implies that Thr383 phosphorylation may be the first step in CHK2 activation. We speculate that the phosphorylation of Thr387 may provide an inhibitory mechanism to curtail activation in its cellular context. This observation was made possible by the unique ability of our genetically encoded pThr/Thr/Ala system to toggle between phosphorylated, non-phosphorylated, and non-phosphorylatable forms of CHK2 at precisely defined positions.

### Multi-level interaction analysis of CHK2 substrates

**Fig. 4:**
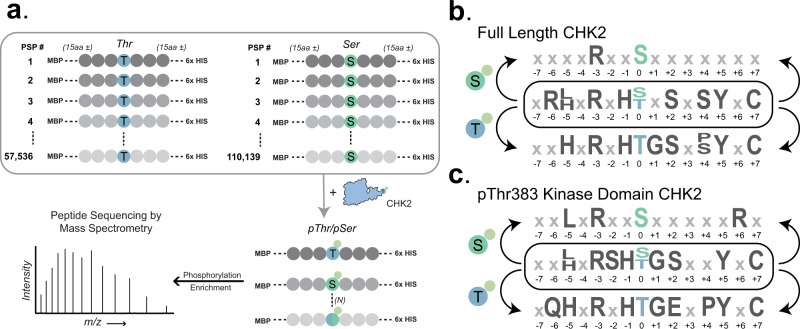
Kinase profiling of CHK2 variants identifies motif elements. **a** Schematic of kinase profiling with active CHK2 kinase against both Ser and Thr phosphosite libraries. pLogo motif enrichment analysis of phosphosite substrates identified by LC-MS/MS for **b** full-length CHK2 and **c** pThr383 CHK2 KD subclassified by Ser (top), Thr (bottom), and Ser/Thr (middle) phosphosite library IDs. Only significant identity elements are shown (*N* = 3, *p* < 0.05 as determined by pLogo).

The regenerable production of active kinases and non-phosphorylated Ser/Thr phosphosite libraries presents an opportunity for the determination of individual kinase substrates across the human proteome with greater resolution and coverage^26^. Our approach relies upon the expression and purification of the non-phosphorylated phosphosite libraries and an active kinase of interest. The kinase is then reacted with the phosphosite library in vitro, enabling a proteome-wide phosphosite substrate screen (Fig. 4a). Reactions are subsequently enriched for phosphopeptides and analyzed by LC-MS/MS. We were previously limited to reactions consisting of Ser phosphosites, but our Thr library expanded the theoretical coverage to ~167,000 potential phosphorylation sites encoded as unique peptides. By coupling purified Ser and Thr phosphosite libraries to in vitro kinase reactions and MS analysis, we could profile the activity of both full-length CHK2 and the activated pThr383 KD (Fig. 4a). Overall, we identified 126 phosphosites in full-length CHK2 and 158 in pThr383-KD, with 95 phosphosites in common across both CHK2 variants (Supplementary Data 2). While motif analysis^31^ of aggregate phosphosites enriched the canonical CHK2 RxxS/T recognition motif, separation of enriched phosphosites into Ser and Thr sub-pools revealed unique motif elements (detailed motifs provided in Supplementary Fig 11) not seen with traditional peptide arrays^40^ (Fig. 4b, c).

**Fig. 5:**
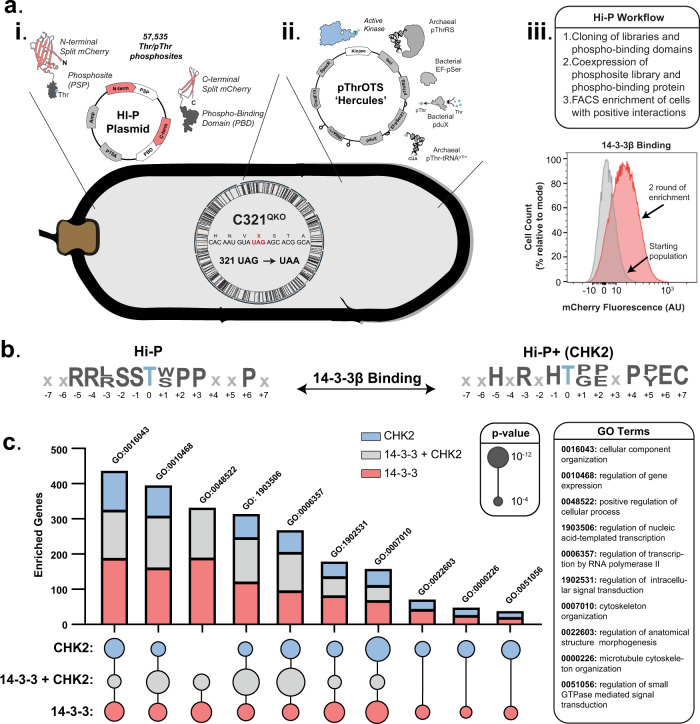
Hi-P+ enables the identification of multi-level, phosphorylation-dependent PPIs. **a** Overview of Hi-P + experimental workflow. (i) Hi-P platform enables phosphorylation-dependent reconstitution of a split mCherry reporter. (ii) Composition of pThrOTS^Hercules^, a modified pThrOTS vector harboring a kinase dependent on pThr-mediated activation (pThr383 CHK2 KD). (iii) Example of 14-3-3β Hi-P analysis and successive rounds of fluorescent (interaction positive) cell enrichment by FACS. **b** Motif analysis of enriched pThr phosphosites interacting with 14-3-3β (left) and Thr-phosphosites interacting with 14-3-3β in the presence of pThr383 CHK2 KD (right). Motif analysis was performed with pLOGO on phosphosites identified across replicates (*N* = 2, *p* < 0.05). **c** PANTHER overrepresentation analysis for GO biological processes across Hi-P (red), Hi-P+ (grey), and kinase profiling (blue) datasets. For each GO term, the number of genes (chart) is compared to term inclusion (circle) and significance (circle size, *p*-value). GO terms listed above each bar are detailed in the grey callout to the right. Significance was determined by PantherDB using Fisher’s Exact test with a Bonferroni correction. Full enrichment terms and *p*-values can be found in the source data file.

Although phosphorylation is a regulatory medium for kinase-phosphatase-dependent processes, it also facilitates non-enzymatic PPIs via interaction with protein-containing phospho-binding domains (PBDs). We had previously developed the Hi-P platform for rapid, high-throughput screening of PBD-phosphosite interactions^8^ (Fig. 5a). The combination of pThrOTS technology with a recombinant human pThr peptide library expands Hi-P to now encompass proteome-wide characterization of pThr-dependent protein interactions. From our Hi-P analysis of 14-3-3β (Supplementary Fig 6D), we found over ~200 unique pThr binding sites across replicates (Supplementary Data 2). Motif analysis of enriched pThr phosphosites showed that pThr Hi-P identified the canonical 14-3-3β binding motif (-3 Arg, -2 Ser, and +2 Pro; Fig. 5b left) we previously identified with pSer Hi-P^8^.

A long-standing barrier to establishing mechanisms of phospho-regulation has been the need for a high-throughput method to connect kinase activity at an individual phosphosite to secondary phosphorylation-dependent PPIs at the same site. To address this challenge, we developed a modified Hi-P framework that identifies kinase-specific substrates interacting with phospho-binding domains across the phosphoproteome (Fig. 5ai, ii). Modification of the Hi-P platform included utilizing the Thr-phosphosite library as the kinase substrate (Thr encoded by ACC), which then leaves the TAG codon free for genetically encoded pThr to activate CHK2. To streamline our approach, we modified pThrOTS^Zeus^ to create pThrOTS^Hercules^ which reduced metabolic burden of heterologous protein expression by changing the origin of replication from ColE1 with Rop to a lower copy ClodF13^41^ and placing CHK2 pThr383-KD under the control of constitutive low-level glnS* transcriptional promoter on the same plasmid as the pThrOTS (Fig. 5a). This reconfiguration is termed Hi-P+ (Hi-P “plus” a kinase of interest). Using Hi-P+, we co-expressed pThr383 activated CHK2 with the Thr-phosphosite substrate library paired with 14-3-3β (Fig. 5a). Hi-P+ enabled a coupled screen where activated CHK2 would phosphorylate substrates and CHK2 dependent PPIs would be identified through binding by 14-3-3β. In addition, overlapping CHK2 substrate and 14-3-3β binding motifs could be deduced. To control for kinase-independent reactions, Hi-P is performed for the PBD of interest (14-3-3β) with both Thr and pThr phosphosite libraries (Supplementary Fig 6A, D) in the absence of kinase expression. This integrated approach identified 409 phosphosite interactions with 14-3-3β that were mediated by CHK2 phosphorylation. Comparing the motifs of CHK2 kinase profiling with 14-3-3β Hi-P and Hi-P+ identified shared substrate and amino acid preference (Figs. 4c, 5b, S13, Supplementary Data 2). Interestingly, a -3 Arg is preferred by both CHK2 and 14-3-3β (Fig. 5b), yet a -1 His was preferred only in CHK2 Thr substrates (Fig. 4c). Hi-P+ demonstrated that the -1 His-pThr motif was recognized by 14-3-3β (Fig. 5b) and highlighted how coupling an active kinase with a direct PPI readout can uncover kinase-specific PPI network criteria. To provide functional context to the results of our Hi-P+ platform, we performed a GO enrichment analysis for biological processes on each unique dataset generated throughout this study (Fig. 5c). Our analysis revealed a high degree of overlap for GO terms related to the cytoskeleton and cellular organization and processes involved in gene regulation^42^. Overall, combining pThrOTS technology for kinase activation, a recombinant human Thr peptide library, and a phosphorylation-dependent PPI reporter uniquely established Hi-P + as a single method for the high-resolution determination of multi-level phospho-dependent PPI networks within the human proteome.

## Discussion

Through systematic investigation of individual OTS components, we developed a pThrOTS variant with robust co-translational insertion of pThr at UAG codons. OTS performance was contingent upon intracellular pThr levels, which are dependent on pduX expression levels. Plasmid copy number and regulation of OTS component expression were central to enhancing OTS performance. Deployment of our newly developed pThrOTS^Zeus^ allowed for the identification of CHK2 activation loop phosphorylation at Thr383 as the essential residue regulating kinase activity. The trans-phosphorylation activity of recombinantly expressed CHK2 made it previously impossible to dissect the independent contributions of specific phosphosites to CHK2 activity in vitro. Our approach introduced the ability to toggle between Thr/pThr/Ala substitutions as a unique way to dissect intricate, multi-layered phosphoregulatory mechanisms. Prior to the co-translational insertion of phospho-amino acids by an OTS, the only practical method to study these mechanisms with sufficient detail was through protein ligation techniques which suffer from limitations on phosphosite position within the protein^43,44^. Together with our pThrOTS, the discovery of a Ala suppressor tRNA provided a means to evaluate the phospho-regulatory mechanisms of CHK2 phosphorylation at Thr387/383 in its canonical activation loop. We found that Ala substitutions were not equivalent to Thr and therefore could not be simply considered equivalent, non-phosphorylated residues. Our approach enables pThr to be directly compared with Thr without the deleterious effects of alanine substitutions. This subtle but important observation may have broader impacts if the approach is applied to other kinases or regulatory phosphorylation sites. These advances demonstrate the general utility of our approach towards the characterization of individual kinase regulatory events, and ultimately, the expansion of our fundamental understanding of phospho-regulatory mechanisms.

Using non-phosphorylated versions of our phosphosite libraries, we can rapidly profile the activity and specificity of Ser/Thr kinases. Leveraging this approach, we identified the phosphosite substrates for full-length CHK2 and CHK2 pThr383-KD, allowing us to identify motif elements overlapping with canonical 14-3-3β binding sites. Until now, the determination of phosphorylation-mediated PPIs has been limited to single protein-substrate interactions. Hi-P + provides cell-type independent platform to link kinases and phospho-binding proteins simultaneously to individual phosphosites in a single experimental approach. Multi-level PPIs identified by Hi-P + pave the way for constructing more comprehensive protein interaction networks by linking kinases and PBDs to individual phosphosites across the human proteome. Our platforms have the potential to dramatically expand our understanding of the physiological roles of protein phosphorylation within the cellular environment.

## Methods

### Cell growth and general techniques

All DNA oligonucleotides were obtained with standard purification and desalting from Keck Oligonucleotide Resource, Yale University. DNA sequencing services were obtained through the Keck DNA Sequencing facility, Yale University. Unless otherwise stated, all cultures were grown at 37 °C in LB-Lennox medium (LB, 10 g/L bacto tryptone, 5 g/L sodium chloride, 5 g/L yeast extract). LB agar plates were LB plus 16 g/L bacto agar. Antibiotics were supplemented for selection, where appropriate (Kanamycin at 50 μg/mL, Spectinomycin at 50 μg/mL, Tetracycline at 12 μg/mL, and Ampicillin at 100 μg/mL). *E. coli* Top10 cells (Invitrogen, Carlsbad, CA) were used for cloning and plasmid propagation. NEBuilder HiFi Assembly Mix and restriction enzymes were obtained from New England BioLabs. Plasmid DNA preparation was carried out with the QIAprep Spin Miniprep Kit (Qiagen). Pre-cast 4–12% (wt/vol) Bis–Tris SDS-PAGE gels were purchased from Bio-Rad. Phos-tag reagent for hand-cast protein gels was purchased from FUJIFILM Wako Chemicals U.S.A. Corporation.

All plasmids were transformed into recipient strains by electroporation. Electrocompetent cells were prepared by inoculating 20 ml of LB with 200 μl of saturated culture and growing at 37 °C until reaching an OD_600_ of 0.4. Cells were harvested by centrifugation at 7441 × *g* for 2 min. at 4 °C. Cells pellets were washed twice with 20 ml ice cold 10% glycerol in deionized water (dH2O). Electrocompetent pellets were resuspended in 100 μl of 10% glycerol in dH2O. Fifty nanograms of plasmid was mixed with 50 μl of resuspended electrocompetent cells and transferred to 0.1 cm cuvettes, electroporated (BioRad GenePulser™, 1.78 kV, 200 Ω, 25 μF), and then immediately resuspended in 600 μl of LB. Transformed cells were recovered at 37 °C for 1 h and 100 μl was subsequently plated on an appropriate selective medium.

### Strain construction

Strains were made using two separate methods. A complete list of strains and their deletion primers can be found in Supplementary Data 1. The initial individual knockout strains used in Fig. 1 and Supplementary Figs 1-3 were generated using Multiplexed Automatable Genome Engineering (MAGE)^45^. Briefly, a C321.ΔA strain containing a temperature-inducible lambda red operon is grown to an OD of 0.4 in 3 ml LB at 34 °C. Cells are then set to shake in a 42 °C water bath for 15 min and are then pelleted by centrifugation at max speed for 30 s at 4 °C, the supernatant is then removed by pipetting. The cells are then resuspended in 1 ml of 10% glycerol on ice. The pelleting and resuspension are repeated a total of 3 times. Following the final pelleting, cells are resuspended in 50 μl 10% glycerol with 1 μl of 10 μM MAGE oligonucleotide. Cells are then transferred to 0.1 cm cuvettes, electroporated (BioRad GenePulser™, 1.78 kV, 200 Ω, 25 μF), and then immediately resuspended in 600 μl of LB. Transformed cells were recovered at 34 °C for 1 h. This process was repeated a total of three times in one day, with the final set of cells being plated on LB plates. Knockouts were confirmed using colony PCR. MAGE primers were designed to produce a frame shift mutation and generate multiple stop codons early in the ORF. MAGE primers were ordered from IDT with enhanced stability modification (denoted as *). All primers used in MAGE are listed in Supplementary Data 1.

All C321.ΔA.exp and BL21 (DE3) based strains were generated by traditional recombineering methods with a FRT-kanamycin-FRT casette^46^. Primers with homology to the gene of interest were amplified from pKD4^46^ to generate a homologous sequence with an internal FRT-kanamycin-FRT casette. Primers used in this study were those previously validated for each knockout in the Keio collection^47^. Cells were grown and transformed by electroporation as stated above, but with ~50 ng of the PCR product in place of plasmid DNA. Cells were then plated on 50 ng/μl kanamycin LB plates and grown overnight at 37 °C. Individual colonies were then screened by PCR for the presence of the kanamycin-FRT deletion cassette or gene targeted for deletion using a three-primer assay that yielded different sized amplicons based on deletion status of the targeted locus. List of primers used for initial screening can be found in Supplementary Data 1. Cells that contained the kanamycin-FRT cassette and were absent the gene of interest were then grown overnight in 5 ml LB containing 50 ng/μl kanamycin in a 15 ml pop-cap (Falcon) tube and set to shake at 230 RPM and 37 °C. The following day cells were then transformed by electroporation, as mentioned above, using the pCP20 plasmid, containing a temperature-sensitive origin and Flp recombinase. Cells were selected on 100 ng/μl ampicillin LB plates and grown overnight at 30 °C. Individual colonies were then patched on separate plates containing either 100 ng/μl ampicillin, 50 ng/μl kanamycin, or no antibiotic in LB. Plates were incubated overnight at 30 °C. Individual colonies that were negative for kanamycin resistance and positive for ampicillin resistance were then streaked on LB plates and incubated overnight at 42 °C. The following day plates were transferred from 42 °C to 30 °C for additional growth. Surviving colonies were then screened by PCR a final time to confirm deletion of the target locus as seen in Supplementary Fig 3. All primers used for the generation of knockout cassette and screening for the gene of interest can be found in Supplementary Data 1.

### Construction of OTS vectors

The detailed construction of OTS plasmids may be found in the history of each plasmid map file in Supplementary Data 8. The starting pThr tRNA and pThrRS were generated by site-directed mutagenesis of a pSerOTS^19^, gifted from the O’Donoghue lab. The pduX gene, OXB20 promoter, and proK tRNA cassette were produced as separate gene gBlocks from GeneWiz. The EF-pSer1 and EF-pSer21 were amplified from previously reported pSerOTSs^9,21^. Generally, OTSs were assembled by Gibson assembly using NEBuilder HiFi DNA Assembly master mix (NEB). The pThrOTS backbone was made de novo by a four fragment Gibson assembly. A single tRNA cassette was assembled into the pThrOTS backbone by Gibson assembly to create pThrOTS^Zeus^. The pThrOTS^Hercules^ variant was created by a three fragment Gibson assembly consisting of the (1) pThrRS/EF-pSer21/pduX operon, (2) ClodF13 spectinomycin pUltra backbone^14^, and (3) CHK2 under control of glnS*.

### Analytical gel and immunoblotting

One hundred micromolar Phos-tag acrylamide (FUJIFILM Wako Chemicals U.S.A. Corporation) within hand-cast 12% acrylamide gels were used to separate phosphorylated reporter proteins. SDS-PAGE gels (4–15% acrylamide, Bio-Rad) and Phos-tag gels were transferred onto PVDF membranes. All gels were visualized by immunoblot. Anti-6xHis immunoblots were performed using 1:2,500 diluted rabbit α-6xHis antibody (PA1-983B, Thermo Fisher Scientific) in 5% w/v milk TBST for 1 h and subsequently washed 3× with TBST. Secondary antibody incubations used 1:10,000 diluted donkey anti-rabbit HRP (711-035-152, Jackson ImmunoResearch) in 5% w/v milk in TBST for 1 h and were subsequently washed 3x in TBST. Protein bands were then visualized using Clarity ECL substrate (Bio-Rad) and an Amersham Imager 600 (GE Healthcare Life Sciences). Densitometry analysis was performed using ImageJ^48^. For quantitative analysis of protein yield, ~0.5 OD equivalents of cell lysate were run on both Phos-tag SDS-PAGE and SDS-PAGE immunoblots alongside a series of 6xHis tagged GFP standards of known concentration. For total protein yield, values were extrapolated based on GFP standard curve intensities. For phospho-protein yield, the total protein yield was adjusted based on the ratio of phosphorylated to the non-phosphorylated product derived from densitometry analysis of immunoblots of the same samples separated by Phos-tag SDS-PAGE.

### MS-READ reporter purification

Frozen *E. coli* cell pellets were thawed on ice. Pellets were lysed by sonication with a lysis buffer consisting of 50 mM Tris-HCl (pH 7.4, 23 °C), 500 mM NaCl, 0.5 mM EGTA, 1 mM DTT, 10% glycerol, 50 mM NaF, and 1 mM Na_3_O_4_V. The extract was clarified with two rounds of centrifugation performed for 20 min at 4 °C and 14,000 × *g*. Clarified lysates were applied to NiNTA metal affinity resin and purified according to the manufacturer’s instructions. Wash buffers contained 50 mM Tris pH 7.5, 500 mM NaCl, 0.5 mM EGTA, 1 mM DTT, 50 mM NaF, 1 mM Na_3_VO_4_ and 20 mM imidazole. Proteins were eluted with wash buffer containing 250 mM imidazole. Eluted protein was subjected to 4 rounds of buffer exchange (20 mM Tris pH 8.0 and 100 mM NaCl) and concentrated using an Amicon Ultra-0.5 ml 30 kDa molecular weight cut-off spin filter (Millipore Sigma #UFC503096).

### CHK2, Thr, and pThr library purification

1.5 L cultures of LB 50 μg/ml Kanamycin, 100 μg/ml Ampicillin were inoculated to OD 0.1 with overnight culture containing C321 cells expressing pThrOTS^Zeus^ (or Ser/Ala tRNA suppressor plasmids) and the expression plasmid (see Supplementary Data 8) containing an MBP-6xHis fusion construct. Strain, expression plasmid, and OTS combinations used for the MBP-fusion proteins used in this study can be found in Supplementary Data 8. Cultures were set to shake at 230 RPM, 37 °C. At OD 0.4, 1.5 L flasks were induced with IPTG and arabinose, and set back at 230 RPM, 37 °C for 4 h. For the pThr and Thr libraries, an additional round of expression was similarly inoculated but grown to an OD of 0.8, induced with IPTG and arabinose, and set at 230 RPM, 20 °C for 20 h. Cultures were pelleted at 6220 × *g* for 5 min, transferred to separate 50 ml falcon tubes and frozen at −80 °C.

Thawed cell pellets were resuspended in 30 ml of lysis buffer (50 mM Tris/HCl pH 7.4, 300 mM NaCl, 1 mM DTT, 1 mg/ml lysozyme, 50 mM NaF, 1 mM NaVO_4_, Roche protease inhibitor tablet, 1 μl benzonase), and incubated at room temperature for 20 min, followed by sonication. The lysates were centrifuged at 45,000 × *g*, 45 min, 4 °C, and the clarified lysate was transferred to a 15 ml falcon tube and centrifuged at 45,000 × *g*, 25 min, 4 °C to remove all remaining insoluble material. The clarified lysate was then purified via a gravity column. Briefly, 6 ml (3 ml bed volume) of NiNTA agarose beads (Qiagen #30210) was pre-equilibrated with lysis buffer before applying cell lysate. Resin was then washed with 100 ml of NiNTA wash buffer (50 mM Tris/HCl pH 7.4, 500 mM NaCl, 500 μM DTT, 50 mM NaF, 1 mM NaVO_4_, 5% glycerol, 10 mM imidazole) before eluting with 20 ml NiNTA elution buffer (50 mM Tris/HCl pH 7.4, 500 mM NaCl, 1 mM DTT, 50 mM NaF, 1 mM NaVO_4_, 250 mM imidazole). Eluted protein was then diluted with 20 ml dilution buffer (20 mM Tris/HCl pH 7.4). 6 mLs (3 ml bed volume) of amylose resin (New England Biolabs #E8021L) was applied to a new column and equilibrated with 50 ml amylose wash buffer (20 mM Tris/HCl pH 7.4, 1 mM EDTA, 150 mM NaCl 50 mM NaF, 1 mM NaVO_4_, 5% glycerol). Diluted protein elution was then applied to amylose column and washed with 100 ml amylose wash buffer before eluting with 15 ml amylose elution buffer (20 mM Tris/HCl pH 7.4, 1 mM EDTA, 150 mM NaCl, 10 mM maltose, 50 mM NaF, 1 mM NaVO_4_, 5% glycerol). Samples were concentrated down using Amicon ultra-15 10 kDa cut-off columns (Millipore Sigma #UFC901008). pThr phosphosite libraries were then frozen at −80 °C. CHK2 kinases were dialyzed in SnakeSkin™ (ThermoFisher #68035) overnight at 4 °C in dialysis buffer #1 (50 mM Tris/HCl pH 7.4, 150 mM NaCl, 5 mM BME, 10% glycerol), then dialyzed for 4 h at 4 °C in dialysis buffer #2 (50 mM Tris/HCl pH 7.4, 150 mM NaCl, 5 mM BME, 50% glycerol) before being frozen at −80 °C.

Purified MBP-fusion Ser/Thr phosphosite protein libraries were incubated with 20 μl (40 units) PreScission protease (GE Healthcare Life Sciences) end-over-end overnight at 4 °C. The cleaved samples were then passed through an Amicon molecular weight cut-off to remove the cleaved MBP and any uncleaved protein. Samples were then concentrated using Amicon Ultra-0.5 3-kDa (Millipore Sigma #UFC500396) molecular weight cut-off columns and buffer exchanged with 10 mM Tris, pH 7.4. Eluted protein was subjected to 4 rounds of buffer exchange (10 mM Tris pH 7.4 and 150 mM NaCl) and concentrated using an Amicon 30 kDa molecular weight cut-off spin filter (Millipore Sigma # UFC503096). Representative Coomassie stained SDS-PAGE gels for the pThr library and a phosphorylated CHK2 KD variant can be found in Supplementary Fig 13.

### Protein digestion and mass spectrometry

#### MS-READ analysis

Affinity purified, buffer exchanged protein was digested and analyzed by mass spectrometry as described previously with some modifications^21^. The concentration of protein was determined by UV280 spectroscopy, 5 μg of MS-READ reporter was dissolved in 12.5 μl solubilization buffer consisting of 10 mM Tris-HCl pH=8.5 (23 °C), 10 mM DTT, 1 mM EDTA and 0.5% acid-labile surfactant (ALS-101, Protea). Samples were heat-denatured for 15 min at 55 °C in a heat block. Alkylation of cysteines was performed with iodoacetamide (IAA) using a final IAA concentration of 24 mM. The alkylation reaction proceeded for 30 min at room temperature in the dark. Excess IAA was quenched with 200 mM DTT and the buffer concentration was adjusted using a 1 M Tris-HCl pH 8.5, resulting in a final Tris-HCl concentration of 150 mM. The reaction was then diluted with water and 1 M CaCl_2_ solution to obtain an ALS-101 concentration of 0.045% and 2 mM CaCl_2_, respectively. Finally, sequencing grade porcine trypsin (Promega) was added to obtain an enzyme/protein ratio of 1/5.3, and the digest was incubated for 15 h at 37 °C without shaking. The digest was quenched with 20% TFA solution resulting in a sample pH of 2. Cleavage of the cleavable acid detergent proceeded for 15 min at room temperature. Digests were frozen at −80 °C until further processing. Peptides were desalted on C_18_ UltraMicroSpin columns (The Nest Group Inc. #SUM SS18V). Columns were initially conditioned with 400 μl 80% ACN 0.1% TFA followed by 200 μl 0.1% TFA. Samples were pelleted at 2000 × *g* for 1 min before having lysate applied to columns. The sample was loaded onto the column and washed twice with 400 μl 0.1% TFA before eluting twice with 200 μl 80% ACN 0.1% TFA. Peptides were dried in a vacuum centrifuge at room temperature. Dried peptides were reconstituted and analyzed by LC-MS/MS. Heatmap values were calculated by normalizing to the minimum or maximum precursor ion intensity relative to pThr intensity. Heatmaps were generated with Plotly^49^ using python 3.8.8^50^.

### Phosphothreonine Phosphosite library digestion, enrichment and dephosphorylation

Five milligrams of purified pThr phosphosite library was digested using S-Trap^TM^ midi columns (Protifi) per manufacturer’s instructions. Phosphoproteins were then enriched using Thermo Scientific High-Select™ Fe-NTA Phosphopeptide-Enrichment Kit (#A32992) followed by Thermo Scientific High-Select™ TiO_2_ Phosphopeptide Enrichment Kit (#A32993) following manufacturer’s instructions. Samples were split into separation fractions before being dried in a vacuum centrifuge at room temperature. Pooled Fe-NTA and TiO_2_ flow-through was cleaned up and desalted using C18 MicroSpin (The Nest Group #SEM SS18V) columns as described earlier. One set of phospho-enriched samples was reconstituted in 2 μl 30% ACN 0.1% FA and vortexed for 30 s. An additional 10 μl of 3:8 70% formic acid: 0.1% TFA was added to each sample and vortexed for 30 s before being spun down. 5 μl was injected for LC-MS/MS analysis.

The remaining dried down phospho-enriched samples were resuspended in 50 μl 1× Protein MetalloPhosphatases (PMP) buffer (NEB) with 10 mM MnCl_2_. Two thousand units of lambda phosphatase (NEB #P0753S) and 5 units calf intestinal phosphatase (NEB #M0525S) were added to each reaction and incubated at 30 °C, 600 rpm, for 1 h in a thermo mixer. Samples were cleaned up using 200 μl StageTips similar to what was previously described^51^. Tips consisted of 2 1.06 mM diameter punches of Empore C18 (3 M #2215) fitted into 200 μ; pipette tips. Columns were conditioned with 40 μl methanol and 30 μl 0.1% TFA. The sample was then acidified with 3 μl 70% FA and vortexed. The sample was applied to the column and centrifuged at 1000 × *g* for 5 min. Columns were washed with 50 μl 0.1% TFA and centrifuged for 3 min at 900 × *g*. Centrifugation was repeated with 20 μl 0.1% TFA. StageTips were then transferred to 2 ml protein low bind tubes (Eppendorf #0030108450) and eluted with 30 μl of 80% ACN 0.1% TFA, centrifuged at 900 × *g* for 1 min. Elution was repeated with 20 μl of 80% ACN 0.1% TFA, centrifuged at 900 × *g* for 2 min. Samples were dried down in a vacuum centrifuge at room temperature. Samples were then reconstituted in in 2 μl 30% ACN 0.1% FA and 10 μl of 3:8 70% formic acid: 0.1% TFA as described earlier.

### CHK2 and threonine phosphosite library digestion

Twenty-four micrograms of cleaved Thr phosphosite library and 5 μg CHK2 kinase were digested using S-Trap^TM^ mini spin columns (Protifi) per manufacturer’s instructions. Following digestions, samples were dried in a vacuum centrifuge at room temperature and frozen at −80 °C.

### Threonine phosphosite library fractionation

Dried down tryptic peptides were fractionated into six fractions using Pierce™ High pH Reversed-Phase Peptide Fractionation Kit (ThermoFisher #84868) following manufacturer’s instructions. Samples were then reconstituted in in 2 μl 30% ACN 0.1% FA and 10 μl of 3:8 70% formic acid: 0.1% TFA as described earlier.

### CHK2 phospho-enrichment

Both full-length CHK2 and CHK2 KD tryptic digests were enriched using Thermo Scientific High-Select™ Fe-NTA Phosphopeptide Enrichment Kit (#A32992) following manufacturer’s instructions. Samples were dried in a vacuum centrifuge at room temperature and reconstituted in 2 μl 30% ACN 0.1% FA and 10 μl of 3:8 70% formic acid: 0.1% TFA as described earlier.

### Data acquisition and analysis

LC-MS/MS was performed using an ACQUITY UPLC M-Class (Waters) and Thermo Q Exactive Plus mass spectrometer. The analytical column employed was a 65-cm-long, 75-μm-internal-diameter PicoFrit column (New Objective) packed in-house to a length of 50 cm with 1.9 μm ReproSil-Pur 120 Å C18-AQ (Dr. Maisch) using methanol as the packing solvent. Peptide separation was achieved using mixtures of 0.1% formic acid in water (solvent A) and 0.1% formic acid in acetonitrile (solvent B) with a 90-min gradient 0/1, 2/7, 60/24, 65/48, 70/80, 75/80, 80/1, 90/1; (min/%B, linear ramping between steps). The gradient was performed with a flow rate of 250 nL/min. A single blank injection (5 μl 2% B) was performed between samples to eliminate peptide carryover on the analytical column. 100 fmol of trypsin-digested BSA or 100 ng trypsin-digested wildtype K-12 MG1655 *E. coli* proteins were run periodically between samples as quality control standards. The mass spectrometer was operated with the following parameters: (MS1) 70,000 resolution, 3e6 AGC target, 300–1700 m/z scan range; (data-dependent-MS2) 17,500 resolution, 1e6 AGC target, top 10 mode, 1.6 m/z isolation window, 27 normalized collision energy, 90 s dynamic exclusion, unassigned and +1 charge exclusion. Data were searched using Maxquant version 1.6.10.43 with Deamidation (NQ), Oxidation (M), and Phospho (STY) as variable modifications and Carbamidomethyl (C) or Dithiomethane (C) as a fixed modification with up to 3 missed cleavages, 6 AA minimum length, and 1% FDR against targeted libraries. *E. Coli*. proteome searches were run against a modified Uniprot *E. coli* database (taken on February 9, 2021) containing MS-READ reporter proteins and OTS components (Supplementary Data 8). Phosphosite libraries were run against a modified FASTA file of the phosphosite library containing a partially digested MBP tag and 6xHis tag (Supplementary Data 4, 5) in MaxQuant with a 1% FDR. Phosphosite libraries were digested in silico using a custom python script and analyzed using modlAMP^30^. MS-READ search results were quantified using Skyline version 20.1.0.31. Proteome search results were analyzed with Perseus version 1.6.2.2 using a two-tailed student’s *t*-test and FDR cut-off of 0.05 to generate volcano plots. Ion spectra were taken from searches using Mascot Daemon 2.5.1. The MS proteomics data have been deposited to the ProteomeXchange Consortium via the PRIDE^52^ partner repository.

### pThr metabolite mass spectrometry

LC-MS-based phospho-amino acid profiling experiment was performed using a Q Exactive plus benchtop orbitrap mass spectrometer equipped with an Ion Max source and a HESI II probe, coupled to a Vanquish UHPLC (Thermo Fisher Scientific, San Jose, CA, USA). The LC-MS experiment was following previously reported methods for mammalian cells^53^, with 1 OD equivalent of *E. coli* cells at mid-log phase pelleted for polar metabolite extraction. The data was analyzed using Xcalibur and GraphPad Prism.

### FACS for Hi-P and Hi-P+

Twenty milliliters of cells containing either no OTS (for Thr libraries), pThrOTS^Zeus^, or pThrOTS^Hercules^ were grown to an OD_600_ of 0.4 and electroporated using the method stated above with either Thr library or pThr library cloned in the split mCherry vector (Supplementary Data 8). The cells were then resuspended in 1.2 mLs of LB and incubated for 1 h at 37 °C and 230 RPM in a 15 ml culture tube. Recovered cells were directly inoculated in 20 ml of LB with 100 ng/μl ampicillin, 50 ng/μl kanamycin and grown overnight at 37 °C and 230 RPM. Cells were plated at 10^-4^ and 10^-5^ serial dilutions on LB plates with antibiotics and grown at 37 °C overnight. Experiments that proceeded forward required at least 20 colony-forming units per 10^−5^ dilution. The following morning, cultures were diluted to an OD_600_ of 0.15 in 5 ml of LB containing either 100 ng/μl ampicillin or 100 ng/μl ampicillin and 50 ng/μl kanamycin grown at 37 °C and 230 RPM. The cells were grown until an OD_600_ between 0.6–0.8 and set on ice. Protein expression was induced using 1 mM IPTG, 0.2% arabinose, and 100 ng/μl anhydrotetracycline, cells were then grown at 20 °C and 230 RPM for 20 h. Thirty microliters of cells were resuspended in 3 ml ice cold PBS in a 5 ml polystyrene tube (Falcon) prior to analysis.

Using a BD FACSAria III running BD FACSDiva 8.0, cells were assessed for mCherry-based fluorescence using a 561-nm laser (Texas Red). Gating was set so that fluorescent enriched cells reached ~200 events/s with a total collection of ~50,000 cells/s. The gating was adjusted for each phospho-binding domain following successive enrichments to ensure that the most fluorescent population was isolated each round. Cells were sorted directly into 3 ml LB without antibiotic, recovered at 37 °C and 230 RPM for 3 h, and then added into 17 ml LB containing antibiotic and grown overnight at 37 °C and 230 RPM (~16 h). The following day sorted cell populations were then used as the starting culture for the second round of protein expression. The procedure for protein expression, preparation for FACS, and cell sorting were repeated, using the same sorting and gating parameters as the first round of sorting. Cells were then recovered, regrown, induced, and prepared for FACS as above. Cellular mCherry fluorescence was then observed using the FACSAria III. Enriched cell populations were visualized using FlowJo 10.6.1. Following the final round of enrichment overnight cultures were pelleted, transferred to a 2 ml Eppendorf tube, and frozen at −80 °C until it was possible to Miniprep the samples using a Qiagen mini-prep kit (#27104). Hi-P experiments were repeated in triplicate during concurrent runs from the same starting population to minimize transformation bias.

### Next-generation sequencing analysis

Sample pellets generated from Hi-P were thawed on ice and Miniprepped (Qiagen #27104) as stated above. 100-200 ng of plasmid DNA was amplified in four reactions using a standardized set of OLS amplification primers (forward: TCTGGGTCGACTGGTGGTACC, reverse: CGTACCATGTAGCTTAATCAGCTGTTAAAGCTT) with Q5 polymerase (NEB #M0491). PCR cycle followed a 98 °C denaturation for 30 s, 15 cycles of 98 °C for 15 s, 58 °C for 30 s, and 72 °C for 30 s, with a final extension of 72 °C for 2 min. Samples were then cleaned and concentrated with a PCR purification kit (Qiagen #28104) to one 50 μl sample. Samples were then further concentrated with a BluePippin (Sage Science) selecting for samples between 100–200 bp. Samples were cleaned and concentrated with a PCR purification kit (Qiagen #28104) to a 40 μl sample. 35 μl of sample at a concentration of 30–50 ng/μl was then sent to Massachusetts General Hospital Center for Computational & Integrative Biology DNA Core for CRISPR amplicon next-generation sequencing.

Sequencing reads were analyzed as reported previously^8^. Briefly, data was first filtered for quality using Trimmomatic, which applied a sliding window filter of width 2 bp and a Phred score cut-off of 30. If the average quality score over two consecutive bases fell below 30, the read was trimmed to remove the remaining bases. Quality trimmed read pairs were then merged using BBMerge with the stringency set to “strict” (https://sourceforge.net/projects/bbmap/). Using custom scripts, the merged reads were then sorted and assigned to the various input libraries based on barcodes added during the PCR amplification step, libraries are provided in Supplementary Data 6, 7. The variable sequence region for each amplicon was then extracted, and for each input library the abundance of every unique sequence was calculated. Sequences with greater than 10× enrichment were taken forward for analysis.

### In vitro kinase reactions

All kinase reactions were carried out in 40 mM HEPES pH 7.5, 20 mM MgCl_2_, 0.1 mg/ml BSA, 50 μM DTT with 200 μM ATP. CHK2 kinase variants tested against CDC25C substrate were carried out by mixing 300 ng of the purified kinase with 3 μg CHKtide in a 15 μl reaction. The initial time point (0 h) was taken before the addition of ATP to the reaction. Following the addition of ATP, reactions were incubated at 30 °C for 1 h shaking at 600 RPM. Reactions were quenched by mixing equal amounts of 2× Laemmli buffer and reaction before freezing at −80 °C. 6 μl of quenched reaction was then run on a 15 lane, 12% acrylamide, 100 μM Phos-tag SDS-PAGE immunoblotted with α-6xHis as described earlier. CHK2 kinase profiling was carried out by mixing 250 ng purified with 3.3 μg cleaved Ser library and 1.7 μg cleaved Thr library in a 40 μl reaction incubated at 30 °C for 4 h shaking at 600 RPM. After 4 h, reactions were frozen at −80 °C before being digested using S-trap mini columns as described earlier. ADP-Glo kinase reactions (Promega #V6930) were carried out per the manufacturer’s instructions using 100 nM kinase and 0.2 μg/μl CHKtide substrate with 100 uM ATP. The assay reaction buffer consisted of 40 mM HEPES pH 7.5, 20 mM MgCl_2_, 0.1 mg/ml BSA, and 50 μM DTT. A standard curve from 0 to 100 μM ADP was used for validation and a no enzyme control was included for background signal subtraction.

### Reporting summary

Further information on research design is available in the Nature Portfolio Reporting Summary linked to this article.

## Supplementary information

Supplementary Information

Description of Additional Supplementary Files

Reporting Summary

Supplementary data 1

Supplementary data 2

Supplementary data 3

Supplementary data 4

Supplementary data 5

Supplementary data 6

Supplementary data 7

Supplementary data 8

## Data Availability

The mass spectrometry proteomics data have been deposited to the ProteomeXchange Consortium via the PRIDE^52^. Datasets can be found under the following accession numbers: PXD026425, PXD026422, PXD026403, PXD026423, PXD026427, PXD026426, PXD026428, PXD026420, PXD026421, PXD026432, PXD037724. NGS files associated with Hi-P and Hi-P+ have been deposited to NCBI, Accession: PRJNA732384 ID: 732384. Materials will be provided upon request. Requests can be made to jesse.rinehart@yale.edu. Source data are provided with this paper.

## Source data

source data

## Code availability

All scripts associated with NGS analysis for Hi-P and Hi-P+ can be found at: https://github.com/rinehartlab/SynPhosphoThreonine.git.
