# Supplementary material for: Enhanced access to the human phosphoproteome with genetically encoded phosphothreonine
Source: Nat Commun. 2022 Nov 24;13:7226. doi: 10.1038/s41467-022-34980-5 (PMC9700786; doi:10.1038/s41467-022-34980-5)
Supplement: Supplementary file 1 — Supplementary Information [file 41467_2022_34980_MOESM1_ESM.pdf]

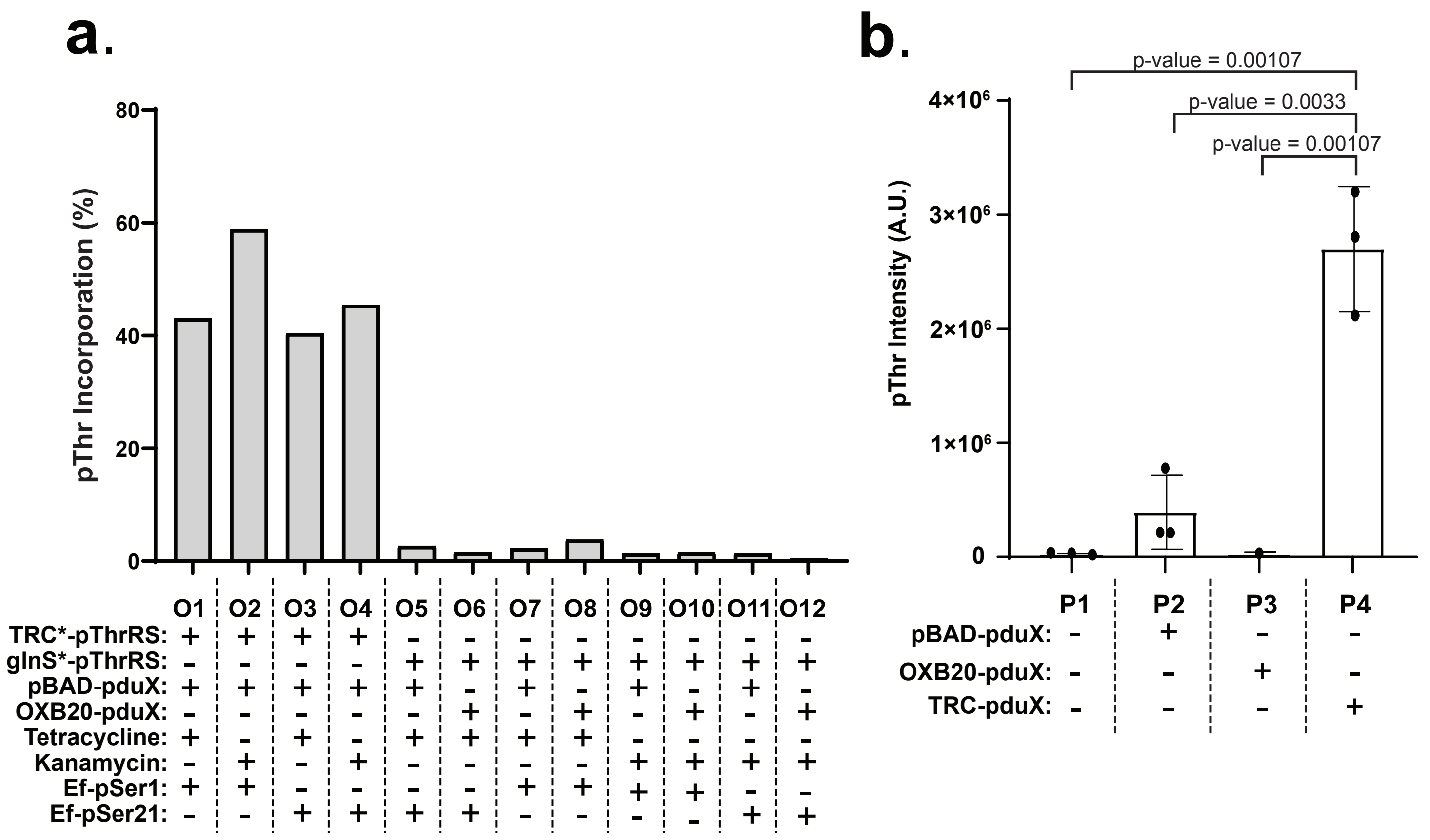

Supplementary Figure 1: Characterization of pThrOTS components. (A) Bar chart displaying pThr incorporation (N=2) for C321ΔserC with pThrOTS variants (O1-O12) expressing the MS-READ reporter. Promoter inclusion denoted by "+" with constitutive promoter activity denoted by "*". (B) Quantification of intracellular pThr levels (N=3, error bars represent +/- 1 S.D.) in C321.ΔserC cells by LC-MS/MS. Significance determined by two-taied student's T-test (equal variance).

a.

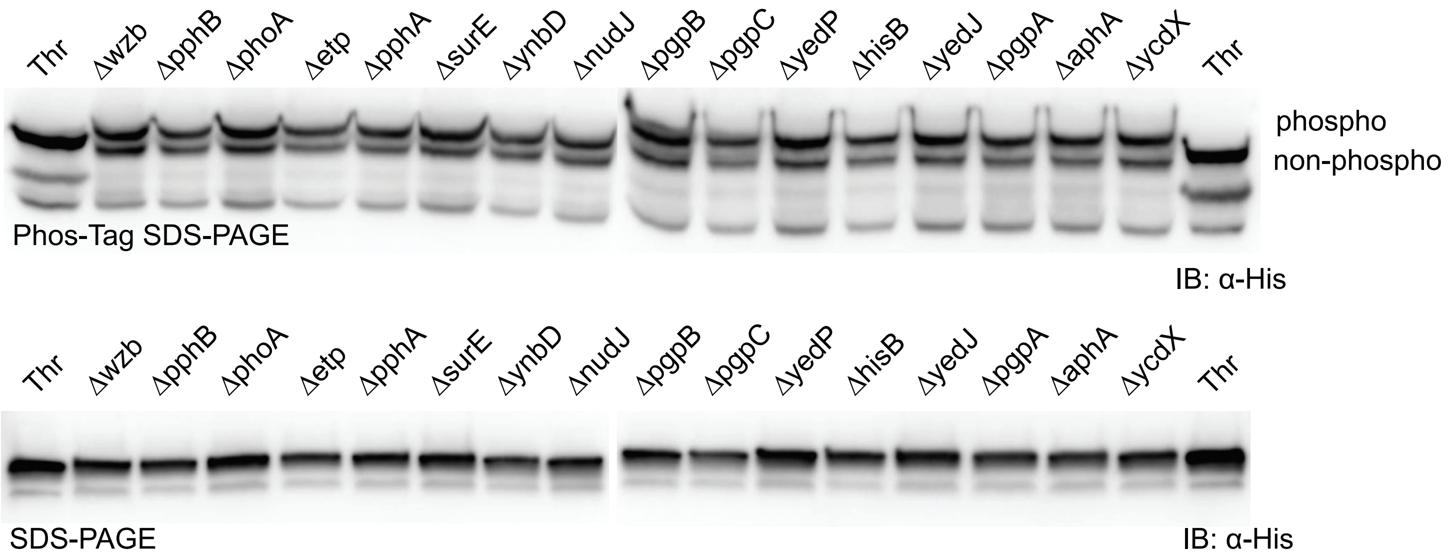

b.

KO	SDS-PAGE	Phospho	Non-Phospho	Phos/Non
wzb	1.29E+04	1.88E+04	1.27E+04	1.47
pphB	1.16E+04	1.56E+04	9.60E+03	1.62
phoA	1.48E+04	2.11E+04	1.21E+04	1.74
etp	1.10E+04	1.60E+04	9.44E+03	1.69
pphA	1.22E+04	1.68E+04	9.24E+03	1.82
surE	1.31E+04	2.03E+04	1.20E+04	1.68
ynbD	1.09E+04	1.69E+04	9.39E+03	1.80
nudJ	1.17E+04	1.77E+04	1.05E+04	1.69
pgpB	1.14E+04	1.84E+04	9.62E+03	1.91
pgpC	9.23E+03	1.19E+04	6.50E+03	1.83
yedP	1.32E+04	1.57E+04	8.23E+03	1.91
hisB	1.12E+04	1.04E+04	5.84E+03	1.78
jedJ	1.27E+04	1.41E+04	7.29E+03	1.93
pgpA	1.09E+04	1.20E+04	7.31E+03	1.64
aphA	1.02E+04	1.25E+04	7.04E+03	1.77
ycdX	1.11E+04	1.33E+04	8.50E+03	1.57

c.

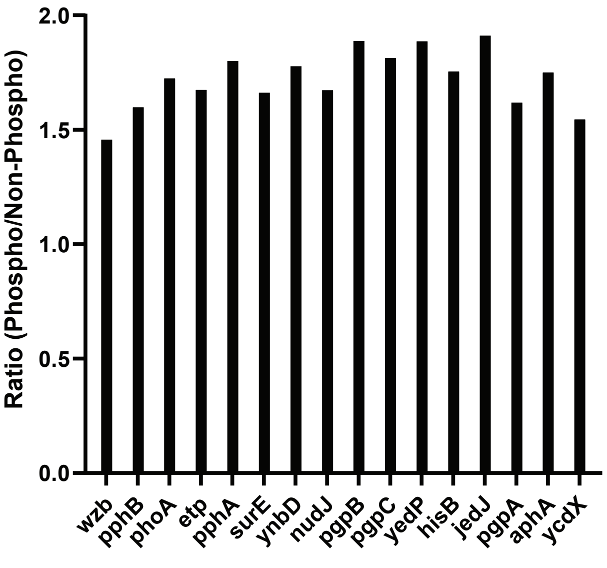

Supplementary Figure 2: Deletion of E. coli phosphatases enhances phosphoprotein production. (A) Analysis of MS-READ reporter phosphorylation and expression using Phos-tag SDS-PAGE (Top) and SDS-PAGE (Bottom) visualized by immunoblot against 6xHis. MS-READ containing Thr (ACC codon) expressed in MG1655 is used as a marker for the non-phosphorylated control band (N=1). (C) ImageJ based densitometry quantification of gel bands in (A) for SDS-PAGE and Phos-tag SDS-PAGE (Phospho & Non-Phospho). (C) Bar graph for the ratio of phosphorylated/non-phosphorylated protein based on Phos-tag SDS-PAGE densitometry.

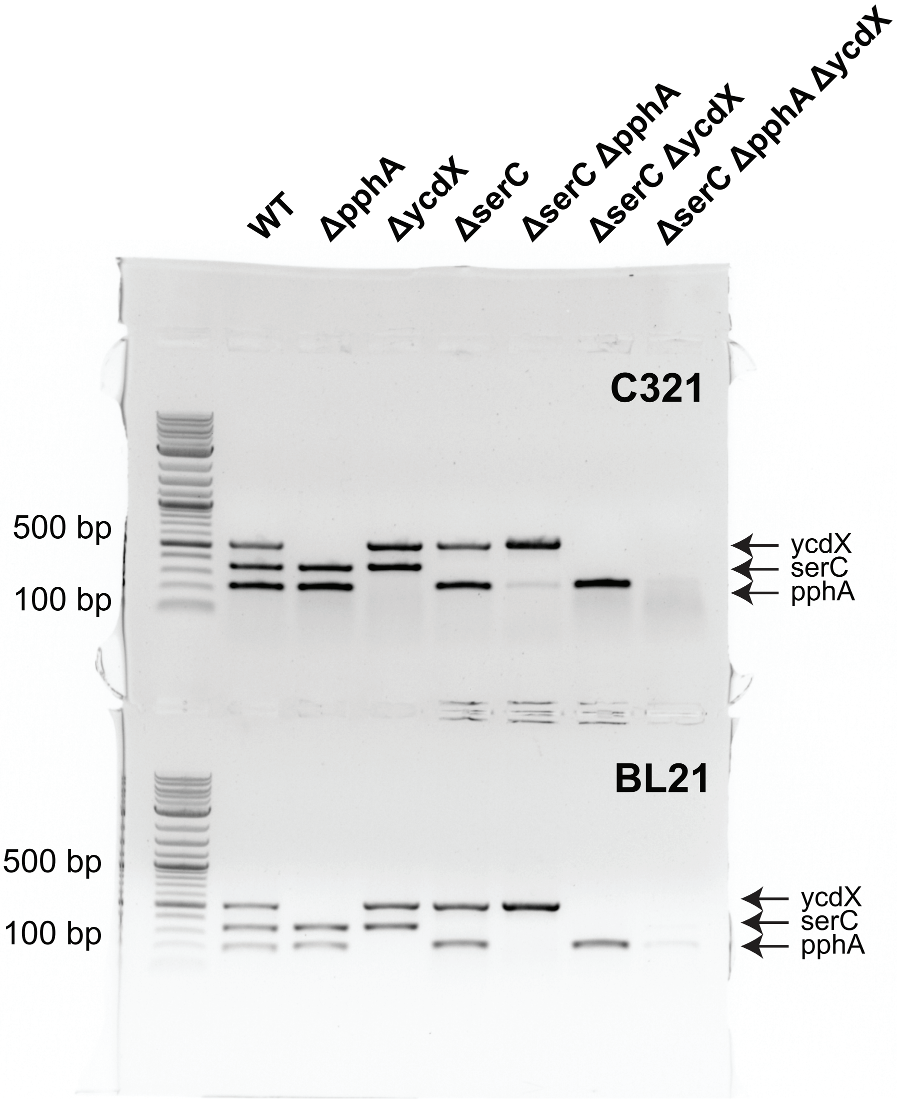

Supplementary Figure 3: PCR validation of chromosomal deletions in E. coli strains. Gene deletion status was assessed by PCR using primer sets targeting pphA, ycdX, and serC. Presence of a band indicates an intact gene product, while absence indicates the gene has been deleted. PCR products were run on a 1% agarose DNA gel and visualized by UV-transillumination (N=1).

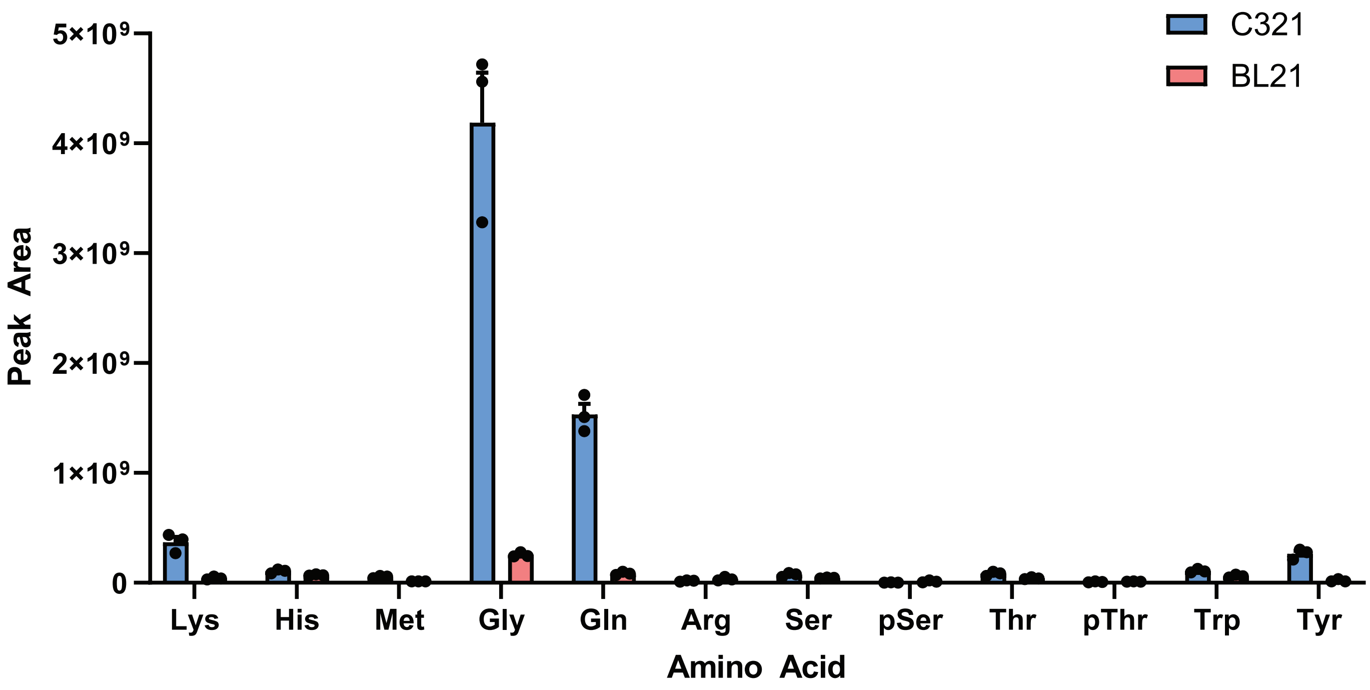

Supplementary Figure 4: Mass spectrometry-based quantification of amino acids incorporated at UAG codons. Purified reporter proteins from C321^{TKO} or BL21^{TKO} expressing tRNA^{pThr} alone were analyzed by mass spectrometry. Identification of peptide misincorporation events were found using MaxQuant. Identified peptides were subsequently quantified by area under the curve (Peak Area) using Skyline, error bars represent +/- 1 S.D., N=3.

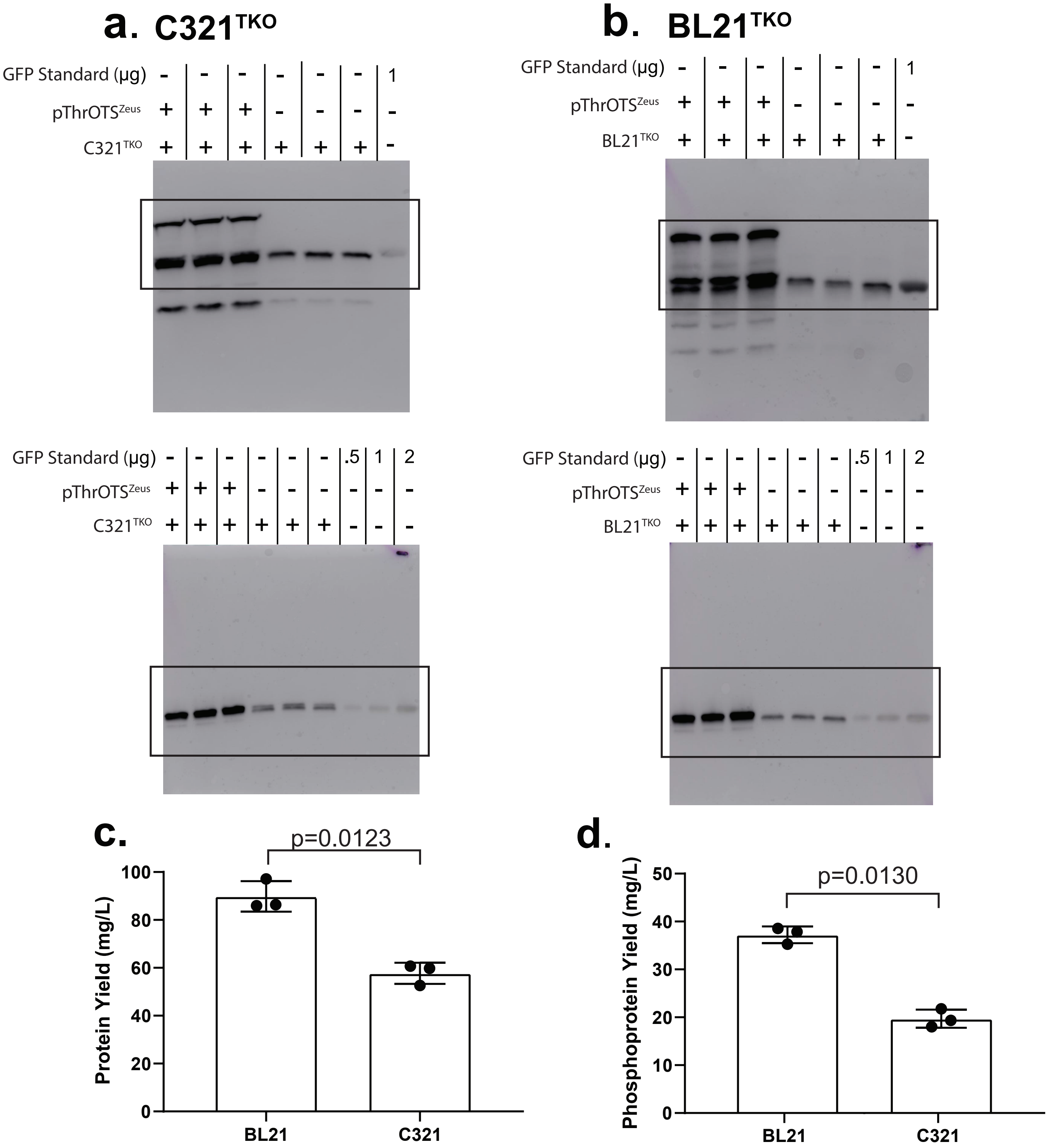

Supplementary Figure 5: Quantification of phospho-protein yield in C321^{TKO} and BL21^{TKO}. Phos-tag SDS-PAGE (top) and SDS-PAGE (bottom) immunoblots for MS-READ protein yield in whole cell lysates for (A) C321^{TKO} and (B) BL21^{TKO}. Boxes denote area used for ImageJ based densitometry. Quantification of (C) total protein and (D) phosphoprotein yield based on immunoblots in (A) and (B). Significance determined by two-taied student's T-test (equal variance), error bars represent 1 S.D., N=3.

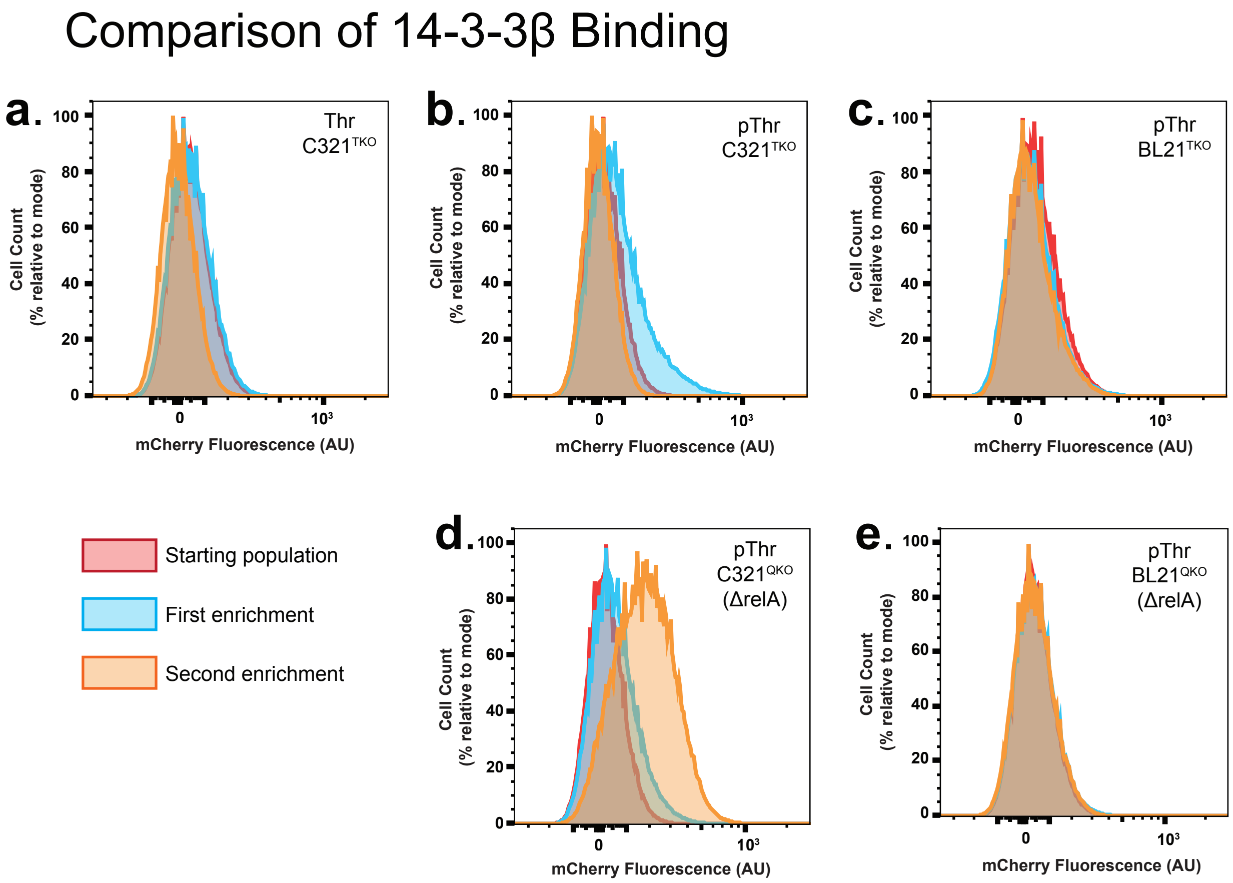

Supplementary Figure 6: Comparison of 14-3-3β Hi-P enrichment for mutliple strains. (A) FloJo overlay control sample for 14-3-3β and the Thr library in C321^{TKO}. Comparison for successive rounds of enrichment for 14-3-3β paired with pThr library and pThrOTS^{Zeus} in **(**B) C321^{TKO} (C) BL21^{TKO} (D) C321^{QKO}(ΔrelA) (E) BL21^{QKO}(ΔrelA) (N=3).

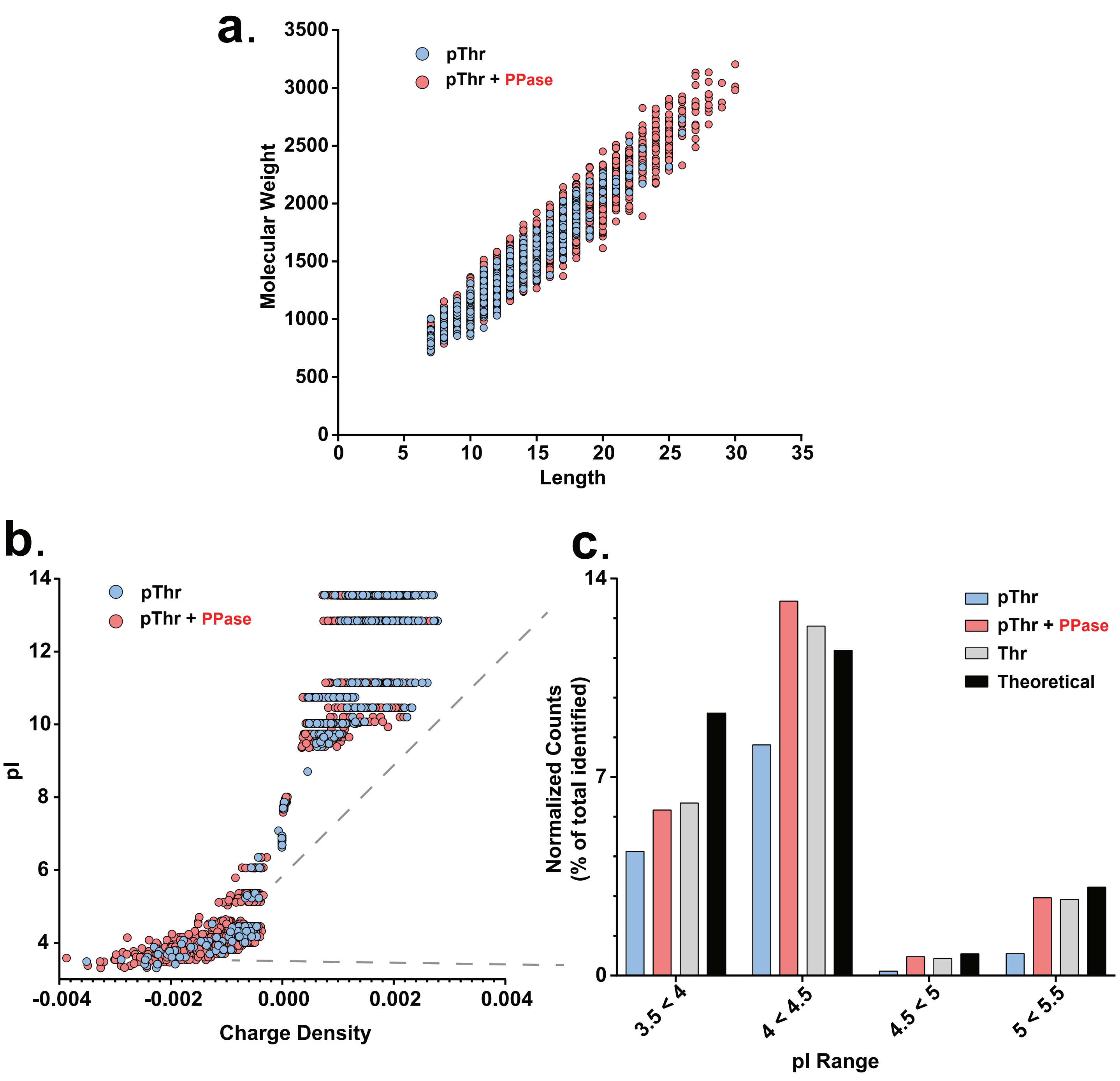

Supplementary Figure 7: Phosphatase treatment alters physicochemical properties of identified peptides. (A) Comparison of length and molecular weight for pThr phosphosites prior to and following phosphatase treatment. Samples outlined in red were enzymatically dephosphorylated (denoted PPase) before LC-MS/MS analysis. (B) Comparison of charge density against isoelectric point (pI) for the pThr phosphosites prior to and following phosphatase treatment. (C) Normalized frequency distributions for the pI of detected phosphosite libraries (blue, red, and grey) compared to a theoretical library with perfect coverage (black).

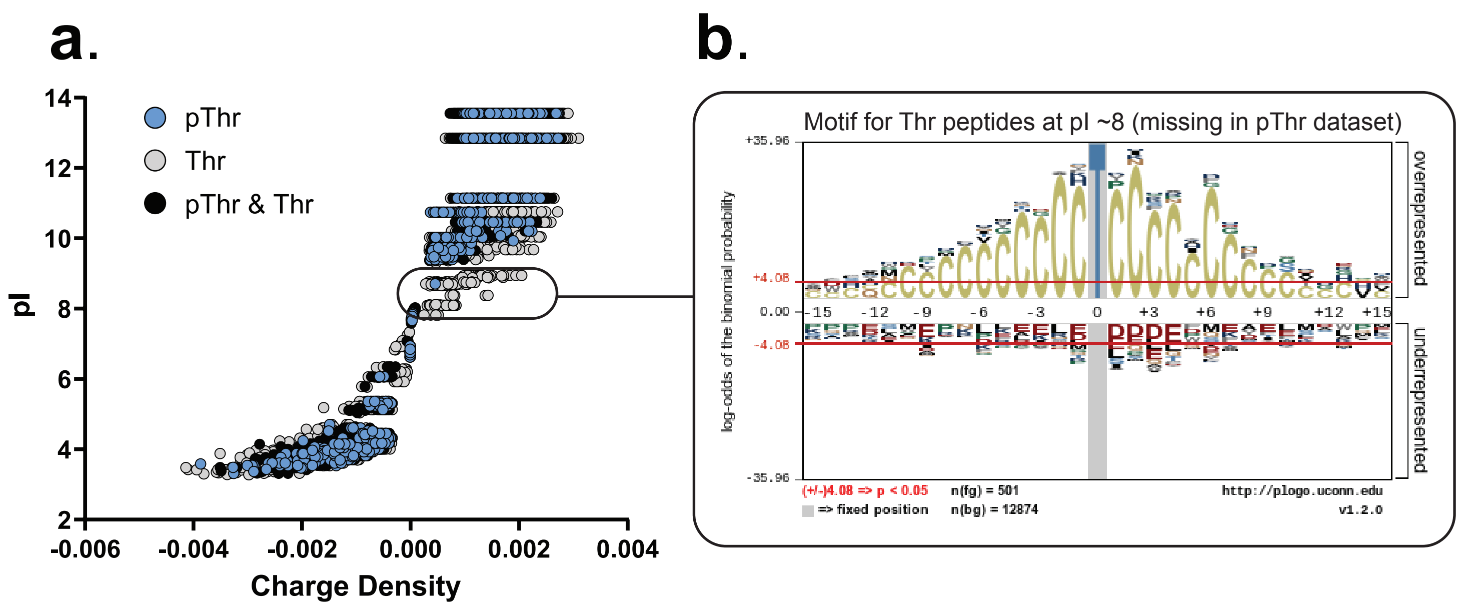

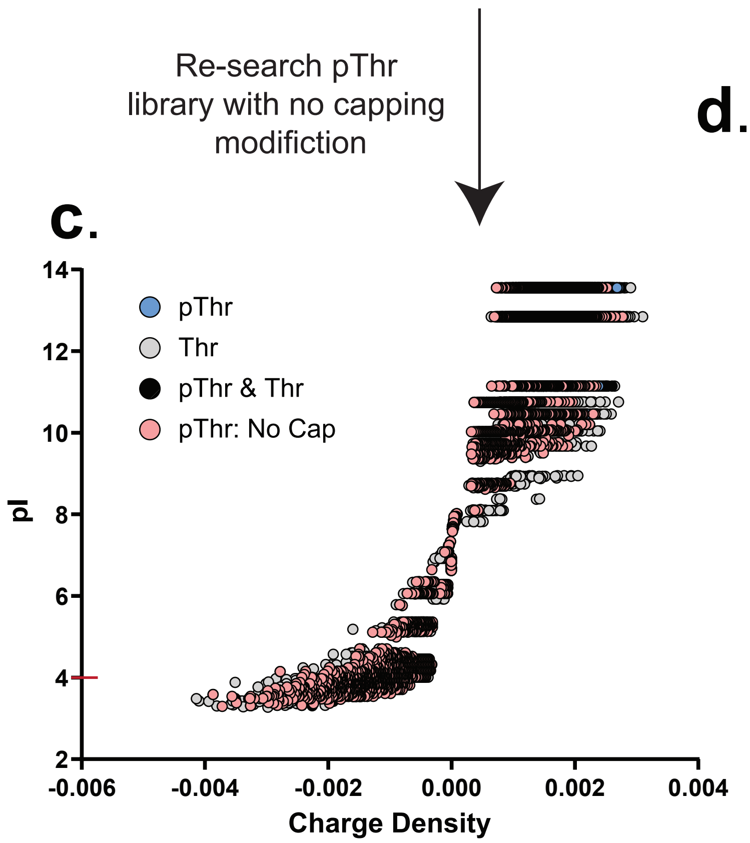

d.

Tryptic Phosphosite Sequence	pI	Phospho-pI
KPGGAVtPQQQQR	10.75	6.78
HtFTLSLPR	9.34	5.80
FNPPSNVtVR	9.23	5.02
VGVHtGTVLGGVLGQK	8.88	6.40
NSSNtLPIPK	8.76	5.06
tLPILTTQAPK	8.66	5.03
HSSGQQNLNTItYETLK	6.11	4.71
AtPSENLVPSSAR	6.04	3.93
IILYENPNFtGK	5.98	3.93
StQLLQAAAAEASLNK	5.68	3.93
tALALEVGELVK	4.51	3.73
GMYDGPVYEVPAtPK	4.35	3.61
TLtGAELELAEAQR	4.23	3.62
NItLDDASAPR	4.19	3.52
TLGVDFIDVAtK	4.19	3.52
SLtELQELEAVYER	4.07	3.53
tPQEYAEGSVEETK	4.07	3.53
GANEDELSPEtSPYVK	3.98	3.46
StDSPGADAELPESAAR	3.90	3.40
SLVLENVtDTAQDIPTTVDTK	3.82	3.34

Supplementary Figure 8: Phosphorylation alters the physiochemical properities of peptides. (A) Charge density plotted against Isoelectric point (pI) for tryptic phosphosite sequences identified across pThr and Thr datasets. (B) Motif (pLogo) analysis for tryptic peptides found in Thr dataset at a pI of 8-9, red lines represent p<0.05. (C) Plot of charge density against pI for the same data from (A) but containing search results for pThr peptides without a capping modification. (D) Representative subset of tryptic phosphosite peptides and their pI values with and without phosphorylation. Cutoff for pI values less than four denoted with a red lie, pThr residues denoted as lowercase in

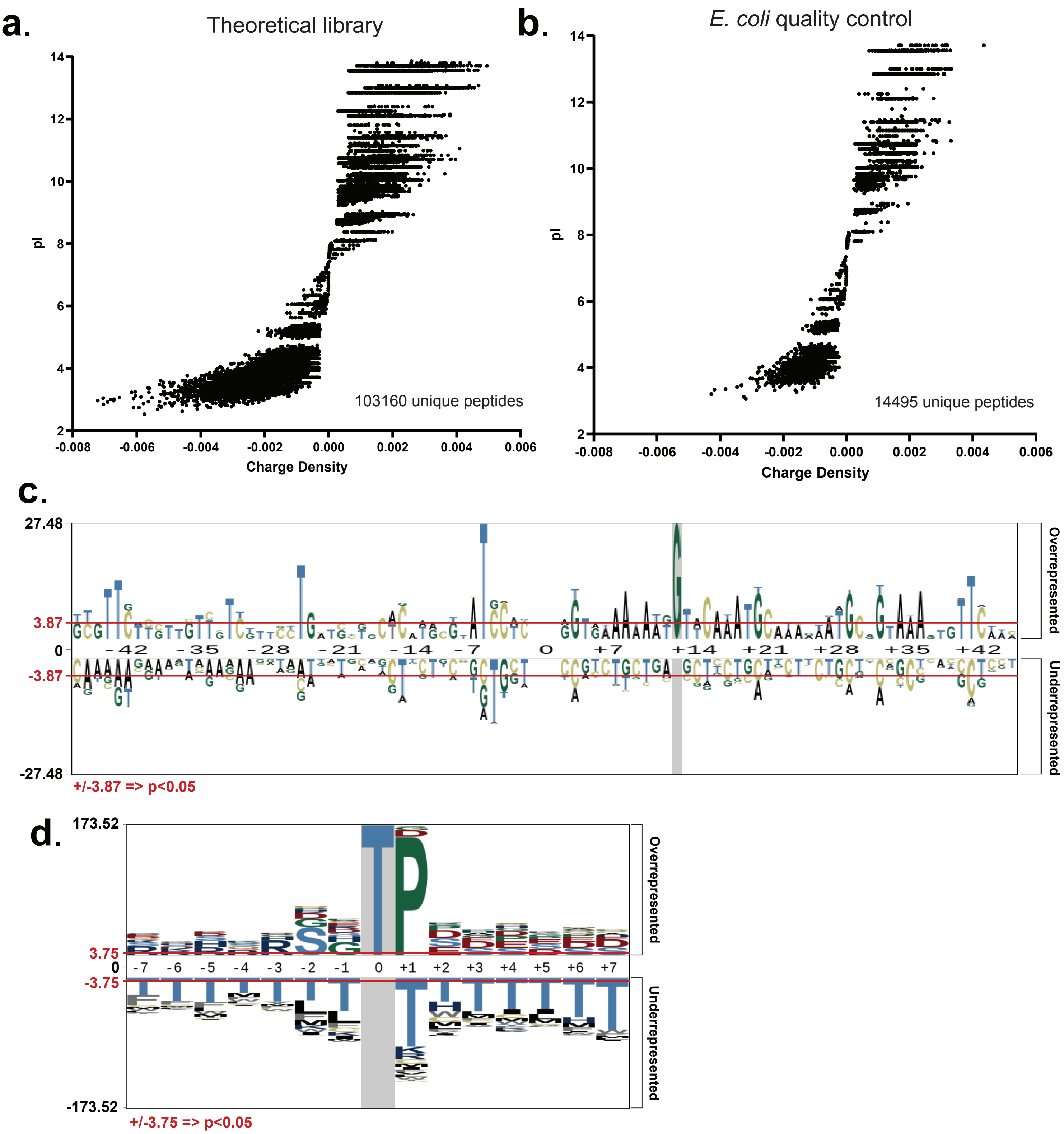

Supplementary Figure 9: Missing data within phosphosite library analysis. Plots for charge density against isoelectric point (pI) for a (A) a representation of the entire theoretical Thr library and (B) Combined *E. coli* quality control samples run before and after phosphosite library runs. pLogo motifs for undetected phosphosites at the (C) DNA level and (D) peptide level, red lines represent $p<0.05$.

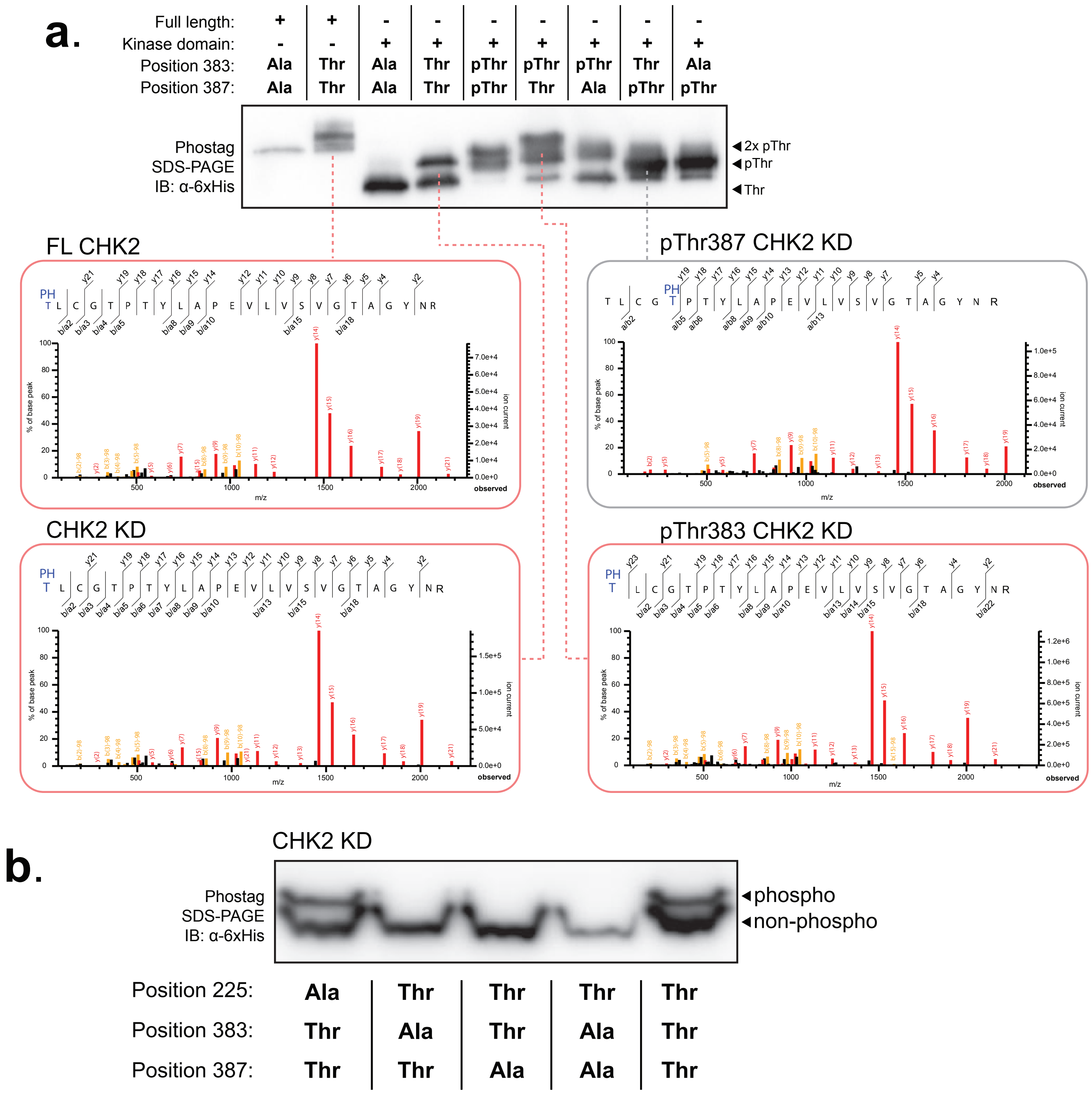

Supplementary Figure 10: Analysis of CHK2 variant phosphorylation status. (A) Immunoblot of phosphorylated CHK2 variants from Figure 4C and corresponding MS/MS ion spectra for CHK2 activation loop peptide fragments obtained from MASCOT. Phosphorylated amino acids denoted by PH (blue, bold) above corresponding residue. Each ion spectra corresponds to samples from a specific lane in (A), with spectra containing pThr383 (green) or pThr387 (red) (N=2). (B) Immunoblot by Phos-tag SDS-PAGE separated CHK2 KD variants expressed in C321^{TKO} with tRNA^{Ala} suppressor facilitated Ala incorporation at residues 225, 383, and 387 (N=2).

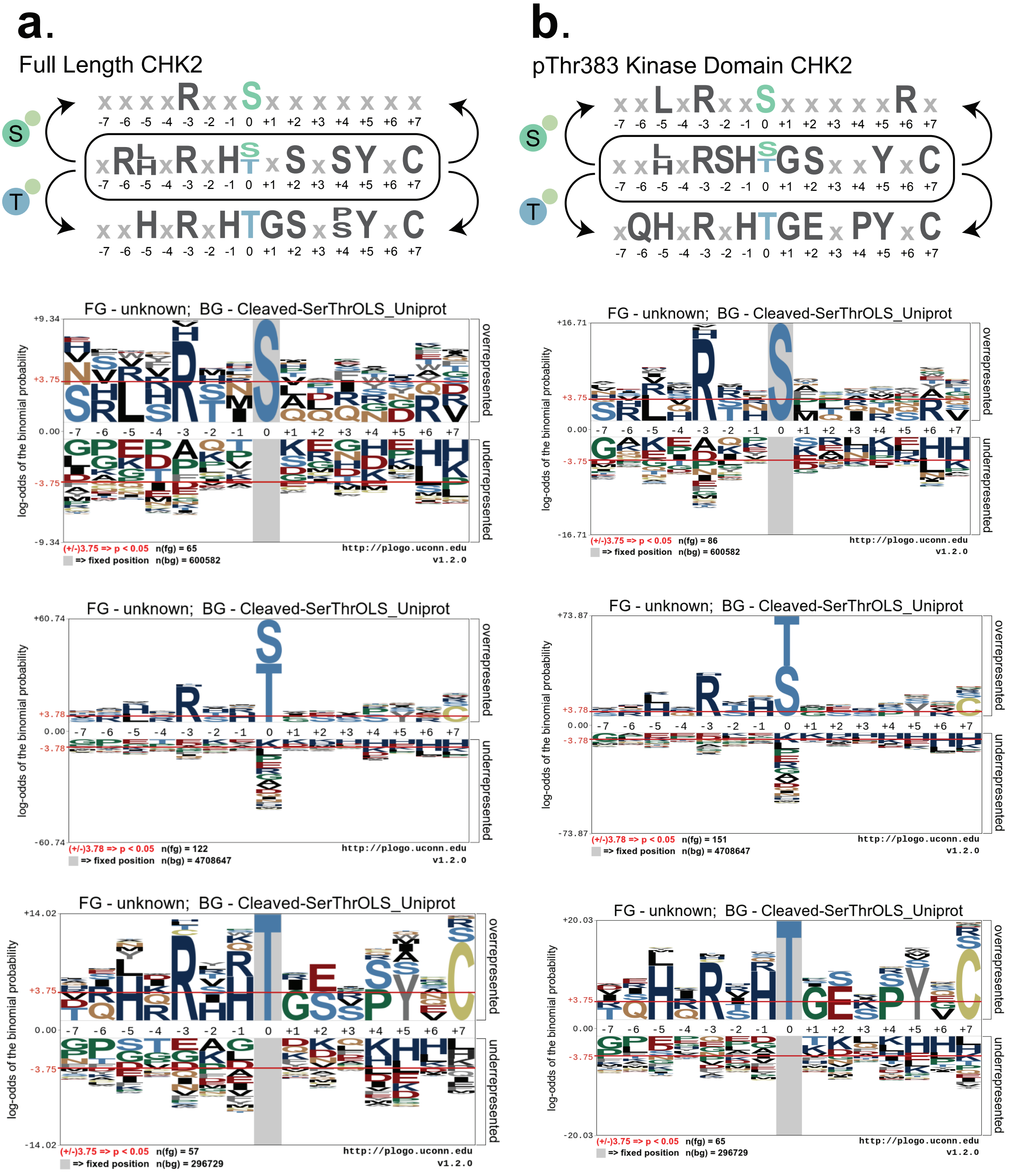

Supplementary Figure 11: Detailed pLOGO motif results for CHK2 kinase profiling. pLOGO motif enrichment analysis of phosphosite substrates identified by LC-MS/MS for (A) full length CHK2 and (B) pThr383 CHK2 KD subclassified by summary (top) Ser (second down), Thr (bottom), and Ser/Thr (second up) phosphosite library. Significant identity elements p<0.05, red lines represent p<0.05.

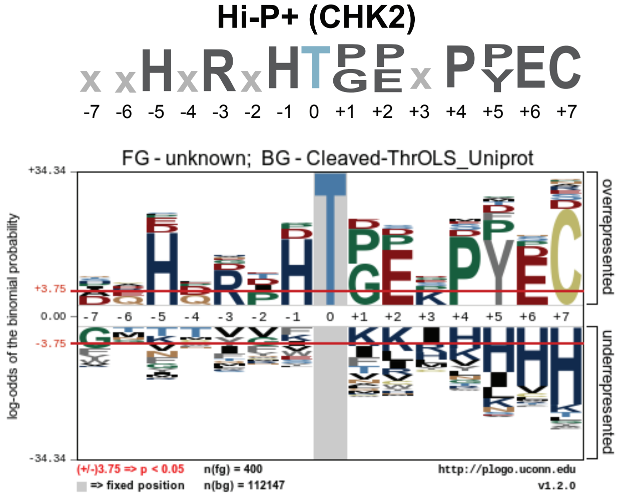

Supplementary Figure 12: pLOGO motif for Hi-P+. Summary motif for HI-P+ with CHK2 and 14-3-3β (top) compared to the generated pLOGO motif (bottom), red lines represent $p<0.05$.

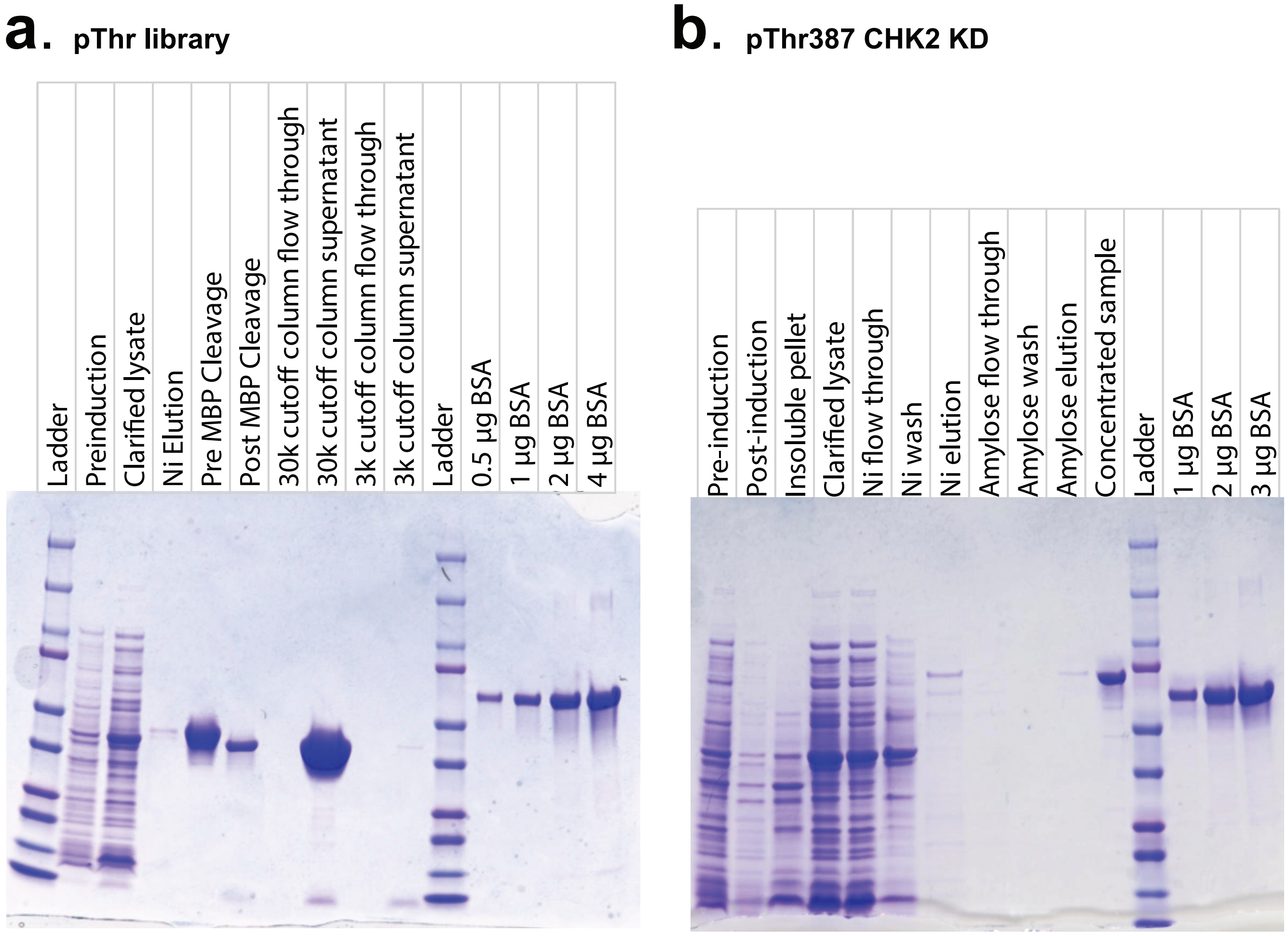

Supplementary Figure 13: Coomassie stained SDS PAGE gels. Protein purification progression for the (A) pThr library and (B) pThr387 CHK2 KD, N=2.
