# Supplementary material for: Enhanced access to the human phosphoproteome with genetically encoded phosphothreonine
Source: Nat Commun. 2022 Nov 24;13:7226. doi: 10.1038/s41467-022-34980-5 (PMC9700786; doi:10.1038/s41467-022-34980-5)
Supplement: Supplementary file 2 — Description of Additional Supplementary Files [file 41467_2022_34980_MOESM2_ESM.pdf]

Title: Supplementary Data 1.
Description: Strain information.

Title: Supplementary Data 2.
Description: Kinase screen and Hi-P data.

Title: Supplementary Data 3.
Description: FASTA files of proteins used and E. coli proteome.

Title: Supplementary Data 4.
Description: FASTA files of Thr phosphosites.

Title: Supplementary Data 5.
Description: FASTA files of Ser phosphosites.

Title: Supplementary Data 6.
Description: Reference DNA sequences for codon optimized Thr phosphosites.

Title: Supplementary Data 7.
Description: Reference DNA sequences for codon optimized Ser phosphosites.

Title: Supplementary Data 8.
Description: Plasmid maps.
