# Supplementary material for: Enhanced access to the human phosphoproteome with genetically encoded phosphothreonine
Source: Nat Commun. 2022 Nov 24;13:7226. doi: 10.1038/s41467-022-34980-5 (PMC9700786; doi:10.1038/s41467-022-34980-5)
Supplement: Supplementary file 3 — Reporting Summary [file 41467_2022_34980_MOESM3_ESM.pdf]

nature portfolio

Corresponding author(s): Jesse Rinehart

Last updated by author(s): Nov 9, 2022

Reporting Summary

Nature Portfolio wishes to improve the reproducibility of the work that we publish. This form provides structure for consistency and transparency in reporting. For further information on Nature Portfolio policies, see our Editorial Policies and the Editorial Policy Checklist.

Statistics

For all statistical analyses, confirm that the following items are present in the figure legend, table legend, main text, or Methods section.

n/a	Confirmed	
☐	☒	The exact sample size (*n*) for each experimental group/condition, given as a discrete number and unit of measurement
☐	☒	A statement on whether measurements were taken from distinct samples or whether the same sample was measured repeatedly
☐	☒	The statistical test(s) used AND whether they are one- or two-sided<br>*Only common tests should be described solely by name; describe more complex techniques in the Methods section.*
☐	☒	A description of all covariates tested
☐	☒	A description of any assumptions or corrections, such as tests of normality and adjustment for multiple comparisons
☐	☒	A full description of the statistical parameters including central tendency (e.g. means) or other basic estimates (e.g. regression coefficient) AND variation (e.g. standard deviation) or associated estimates of uncertainty (e.g. confidence intervals)
☐	☒	For null hypothesis testing, the test statistic (e.g. *F*, *t*, *r*) with confidence intervals, effect sizes, degrees of freedom and *P* value noted<br>*Give P values as exact values whenever suitable.*
☒	☐	For Bayesian analysis, information on the choice of priors and Markov chain Monte Carlo settings
☒	☐	For hierarchical and complex designs, identification of the appropriate level for tests and full reporting of outcomes
☒	☐	Estimates of effect sizes (e.g. Cohen's *d*, Pearson's *r*), indicating how they were calculated

Our web collection on statistics for biologists contains articles on many of the points above.

Software and code

Policy information about availability of computer code

Data collection	BD FACSDiva 8.0<br>Xcalibur 4.3
Data analysis	MaxQuant 1.6.10.43<br>Perseus 1.6.2.2<br>Mascot Daemon 2.5.1<br>FlowJo 10.6.1<br>R Version 4.0.5<br>RStudio 1.4.1103<br>heatmaply 1.2.1<br>GraphPad Prism 8.0.0<br>Python 3.8.8<br>Library data analysis: https://github.com/rinehartlab/SynPhosphoThreonine.git

For manuscripts utilizing custom algorithms or software that are central to the research but not yet described in published literature, software must be made available to editors and reviewers. We strongly encourage code deposition in a community repository (e.g. GitHub). See the Nature Portfolio guidelines for submitting code & software for further information.

Data

Policy information about availability of data

All manuscripts must include a data availability statement. This statement should provide the following information, where applicable:

- Accession codes, unique identifiers, or web links for publicly available datasets
- A description of any restrictions on data availability
- For clinical datasets or third party data, please ensure that the statement adheres to our policy

Mass spectrometry proteomics data have been deposited to the ProteomeXchange Consortium via PRIDE. Data will be published when there is associated DOI or PubMed ID for the work.
Figure 1, Accession: PXD026423
Figure 2/Figures S7-S9, Accession: PXD037724 and PXD026426
Figure 3, Accession: PXD026428
Figure 4/Figure S11, Accession: PXD026420
Figure S1, Accession: PXD026425
Figure S4, Accession: PXD026421
Figure S10, Accession: PXD026432

FASTA files associated MS searches can be found in supplementary data. Peptide library FASTAs were generated in house, E coli proteome was taken from Uniprot reviewed IDs.

NGS files associated with Hi-P and Hi-P+ have been deposited to NCBI, Accession: PRJNA732384 ID: 732384.

Human research participants

Policy information about studies involving human research participants and Sex and Gender in Research.

Reporting on sex and gender	N/A
Population characteristics	N/A
Recruitment	N/A
Ethics oversight	N/A

Note that full information on the approval of the study protocol must also be provided in the manuscript.

Field-specific reporting

Please select the one below that is the best fit for your research. If you are not sure, read the appropriate sections before making your selection.

☒ Life sciences ☐ Behavioural & social sciences ☐ Ecological, evolutionary & environmental sciences

For a reference copy of the document with all sections, see nature.com/documents/nr-reporting-summary-flat.pdf

Life sciences study design

All studies must disclose on these points even when the disclosure is negative.

Sample size	For MS studies our standard sample size was N of 3, as the experiments are time and resource intensive, but this N provides enough information for statistical analysis. A lower N (N of 2) was used in one of the expression control experiments for Thr (not pThr) library expression (Figure 2), and Figure S1A. Smaller N was chosen for Thr library expression because this experiment was an OTS-independent qualitative assessment of hard-coded Thr library coverage with no associated statistical analysis or claims of significance. Similarly, the N for S1A was chosen as a pre-filtering measure by qualitative comparison of performance across a large parameter set. Follow-up experiments were performed on a subset of samples identified through the screening process with N of 3 for statistical comparison. Hi-P and Hi-P+ experiments were performed in duplicate due to practical limitations, and the qualitative application of the data. We directly acknowledge the divergent N numbers for these data and provide justification in the main text.
Data exclusions	No data was excluded.
Replication	All experiments were replicated an additional 1-2 times (as dictated by N numbers), with experiments being carried out on different days with different starting materials to ensure reproducibility.
Randomization	Randomization was not necessary for our project as we were not comparing treatment conditions or biological samples.

Blinding	Blinding was not necessary for our project as we were not comparing any treatments or working with patients/animals that could be influenced by our observations.

Reporting for specific materials, systems and methods

We require information from authors about some types of materials, experimental systems and methods used in many studies. Here, indicate whether each material, system or method listed is relevant to your study. If you are not sure if a list item applies to your research, read the appropriate section before selecting a response.

Materials & experimental systems

n/a	Involved in the study
☐	☒ Antibodies
☒	☐ Eukaryotic cell lines
☒	☐ Palaeontology and archaeology
☒	☐ Animals and other organisms
☒	☐ Clinical data
☒	☐ Dual use research of concern

Methods

n/a	Involved in the study
☒	☐ ChIP-seq
☐	☒ Flow cytometry
☒	☐ MRI-based neuroimaging

Antibodies

Antibodies used	Rabbit anti-6x His antibody (PA1-983B, Thermo Fisher Scientific)<br>Donkey anti-rabbit HRP (711-035-152, Jackson ImmunoResearch)
Validation	Per the manufacturer's website, Thermo Fisher Scientific validated rabbit anti-6xHis antibody using positope (R90050, Thermo Fisher Scientific), a 53 kDa recombinant protein consisting of multiple epitope tags, which has been used as a positive control for 6x-His tag detection.<br><br>Per the manufacturer's website, Jackson ImmunoResearch has verified the antibody reacts via immunoelectrophoresis and/or ELISA with whole molecule rabbit IgG. No antibody was detected against non-immunoglobulin serum proteins. The antibody has been tested by ELISA and/or solid-phase adsorbed to ensure minimal cross-reaction with bovine, chicken, goat, guinea pig, syrian hamster, horse, human, mouse, rat and sheep serum proteins, but it may cross-react with immunoglobulins from other species.

Flow Cytometry

Plots

Confirm that:

☒ The axis labels state the marker and fluorochrome used (e.g. CD4-FITC).

☒ The axis scales are clearly visible. Include numbers along axes only for bottom left plot of group (a 'group' is an analysis of identical markers).

☒ All plots are contour plots with outliers or pseudocolor plots.

☒ A numerical value for number of cells or percentage (with statistics) is provided.

Methodology

Sample preparation	20 ml of cells containing either no OTS (for Thr libraries), pThrOTSZeus, or pThrOTSHercules were grown to an OD600 of 0.4 and electroporated using the method stated above with either Thr library or pThr library cloned in the split mCherry vector (see supplemental plasmid files). The cells were then resuspended in 1.2 mLs of LB and incubated for 1 h at 37°C and 230 RPM in a 15 ml culture tube. Recovered cells were directly inoculated in 20 ml of LB with 100 ng/µl ampicillin, 50 ng/µl kanamycin and grown overnight at 37°C and 230 RPM. Cells were plated at 10-4 and 10-5 serial dilutions on LB plates with antibiotics and grown at 37°C overnight. Experiments that proceeded forward required at least 20 colony-forming units per 10-5 dilution. The following morning, cultures were diluted to an OD600 of 0.15 in 5 ml of LB containing either 100 ng/µl ampicillin or 100 ng/µl ampicillin and 50 ng/µl kanamycin grown at 37°C and 230 RPM. The cells were grown until an OD600 between 0.6–0.8 and set on ice. Protein expression was induced using 1 mM IPTG, 0.2% arabinose, and 100 ng/µL anhydrotetracycline, cells were then grown at 20°C and 230 RPM for 20 hours. 30 µl of cells were resuspended in 3 ml ice cold PBS in a 5 ml polystyrene tube (Falcon) prior to analysis.
Instrument	BD FACSArea III
Software	BD FACSDiva 8.0<br>FlowJo 10.6.1
Cell population abundance	Data was collected on 1,000,000 cells positive for mCherry. Negative proportions varied by sample.

Gating strategy

Gating was set so that fluorescent enriched cells reached ~200 events/sec with a total collection of ~50,000 cells. We do not provide a standard gating figure as the gating strategy is dynamic based on the maximum fluorescence intensity from reconstituted individual protein interactions, which varies based on the identity and recombinant expression level of the constituents (illustrated in Supplemental Figure 6).

- [x] Tick this box to confirm that a figure exemplifying the gating strategy is provided in the Supplementary Information.
