# Supplementary material for: Enhanced access to the human phosphoproteome with genetically encoded phosphothreonine
Source: Nat Commun. 2022 Nov 24;13:7226. doi: 10.1038/s41467-022-34980-5 (PMC9700786; doi:10.1038/s41467-022-34980-5)
Supplement: Supplementary file 12 — source data [file 41467_2022_34980_MOESM12_ESM.zip › Original and Replicate blots revised.pptx]

## Slide 1
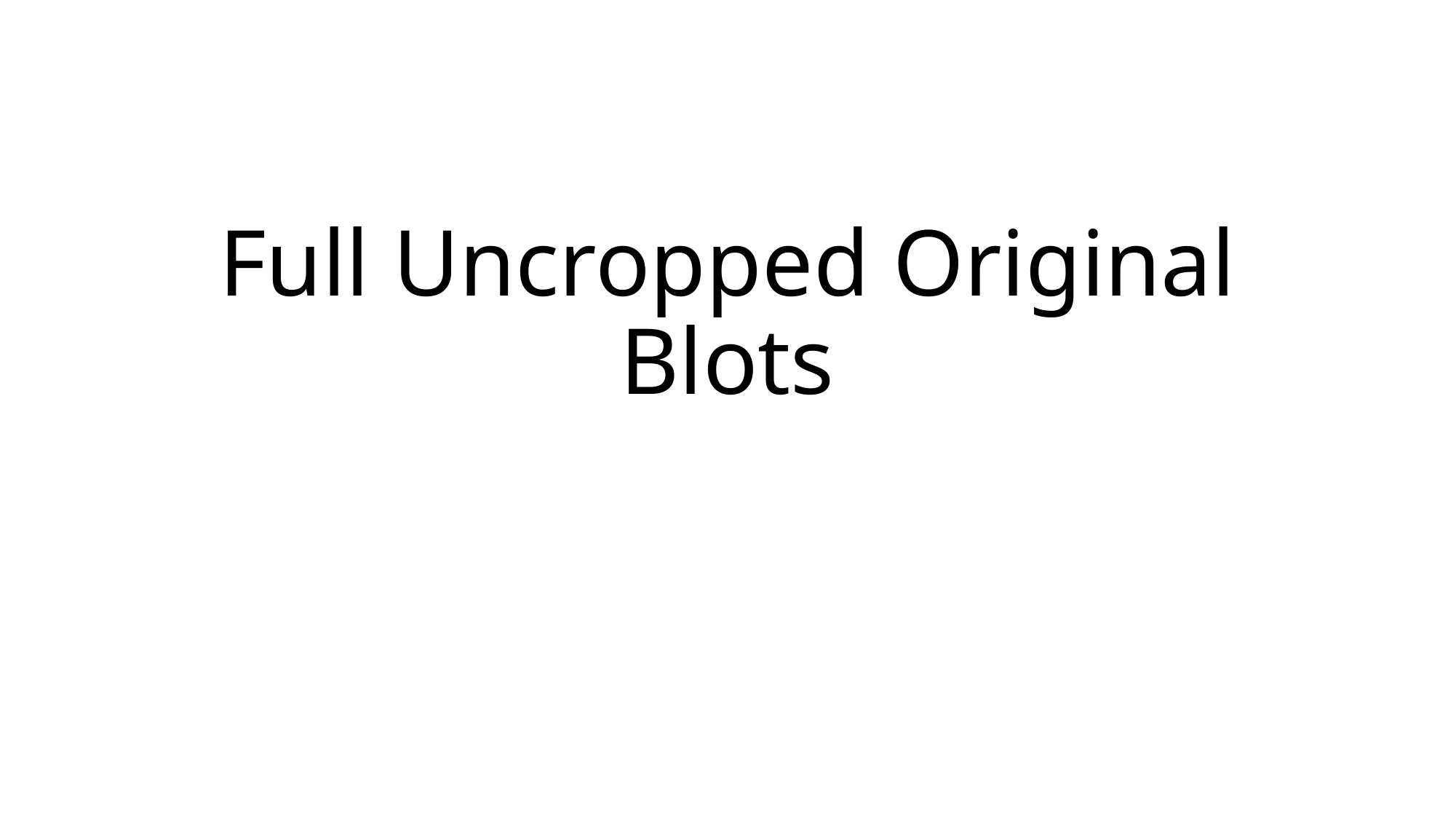

# Full Uncropped Original Blots

## Slide 2
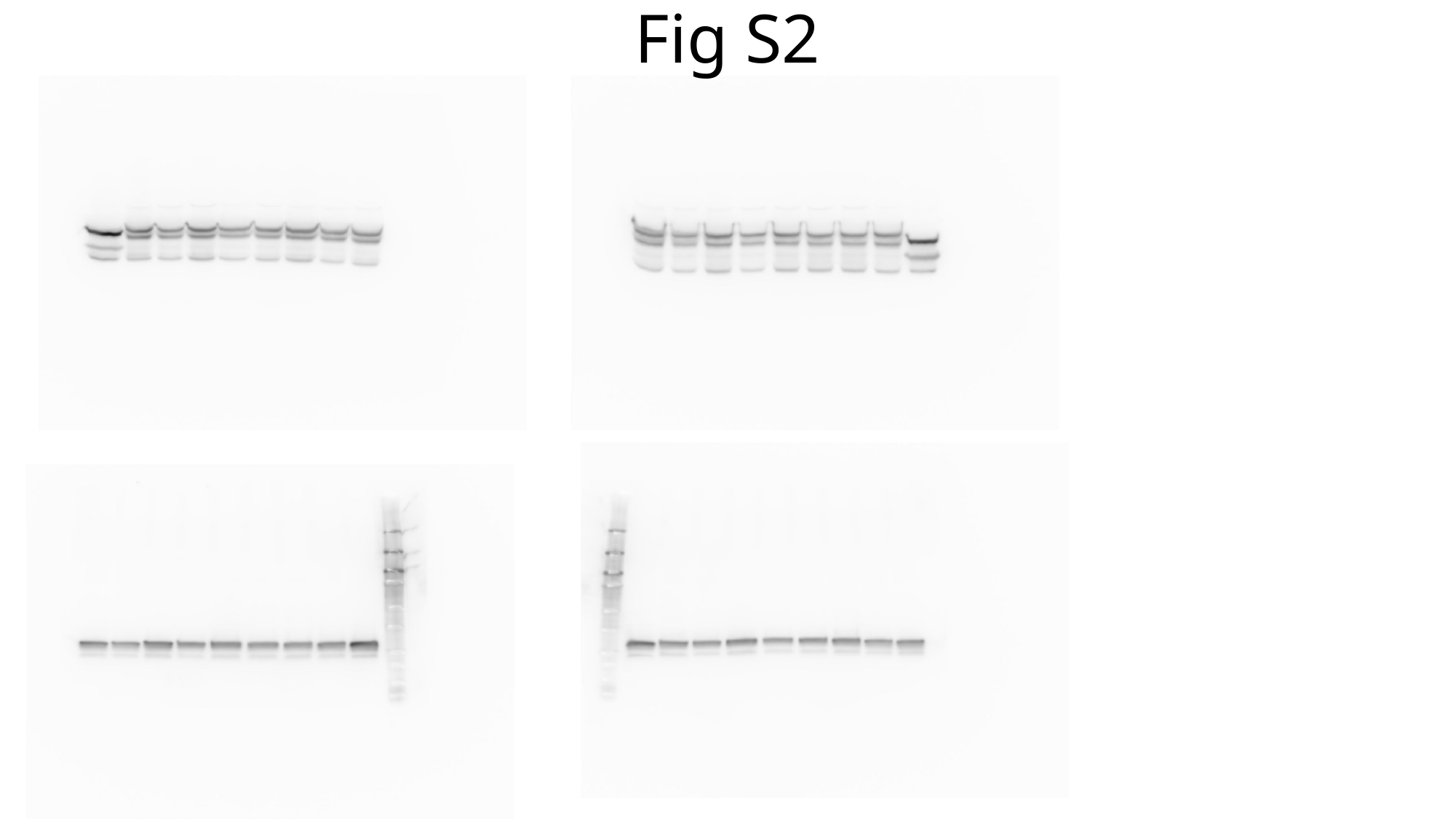

Fig S2

## Slide 3
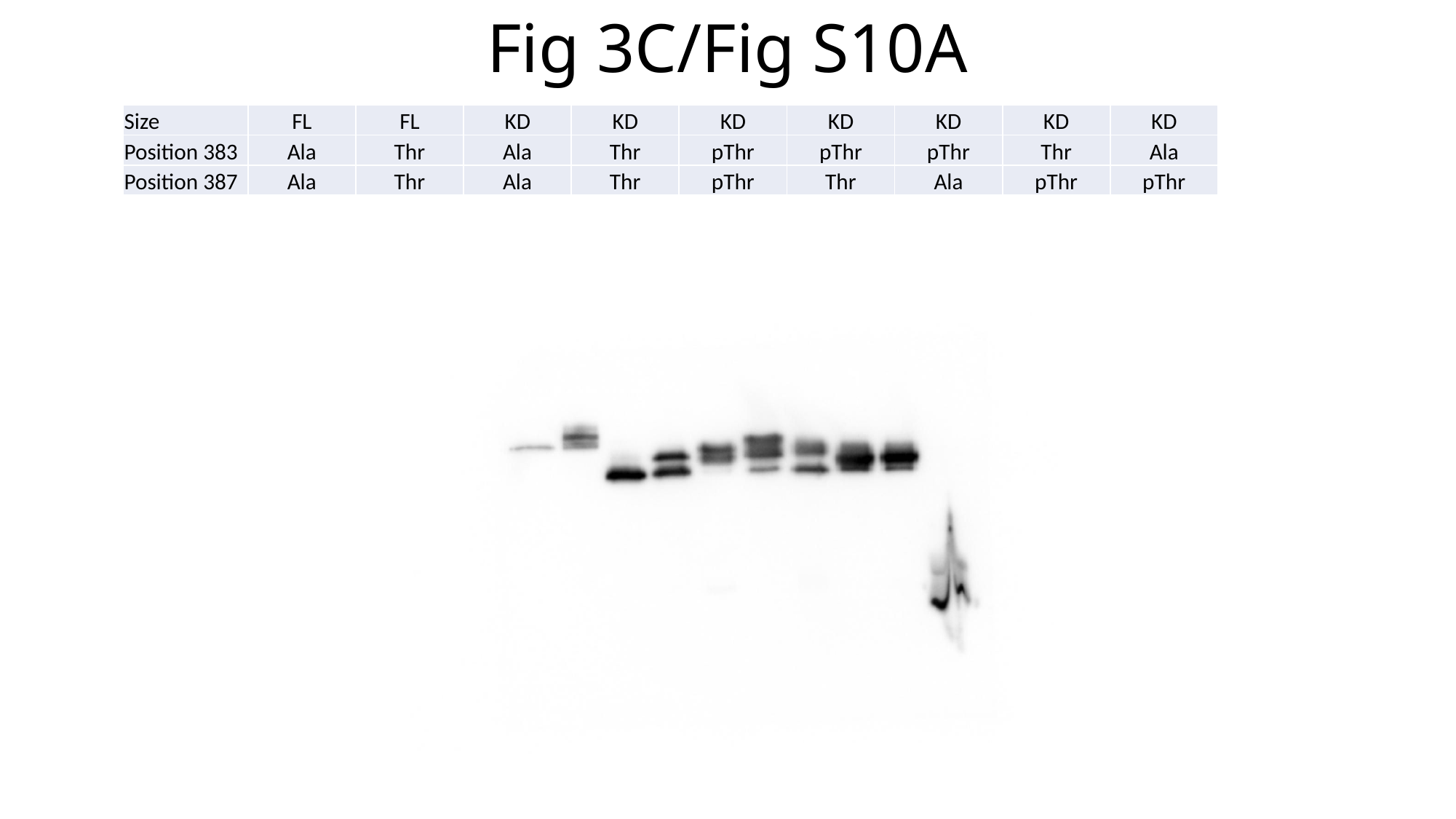

# Fig 3C/Fig S10A
| Size | FL | FL | KD | KD | KD | KD | KD | KD | KD |
| --- | --- | --- | --- | --- | --- | --- | --- | --- | --- |
| Position 383 | Ala | Thr | Ala | Thr | pThr | pThr | pThr | Thr | Ala |
| Position 387 | Ala | Thr | Ala | Thr | pThr | Thr | Ala | pThr | pThr |

## Slide 4
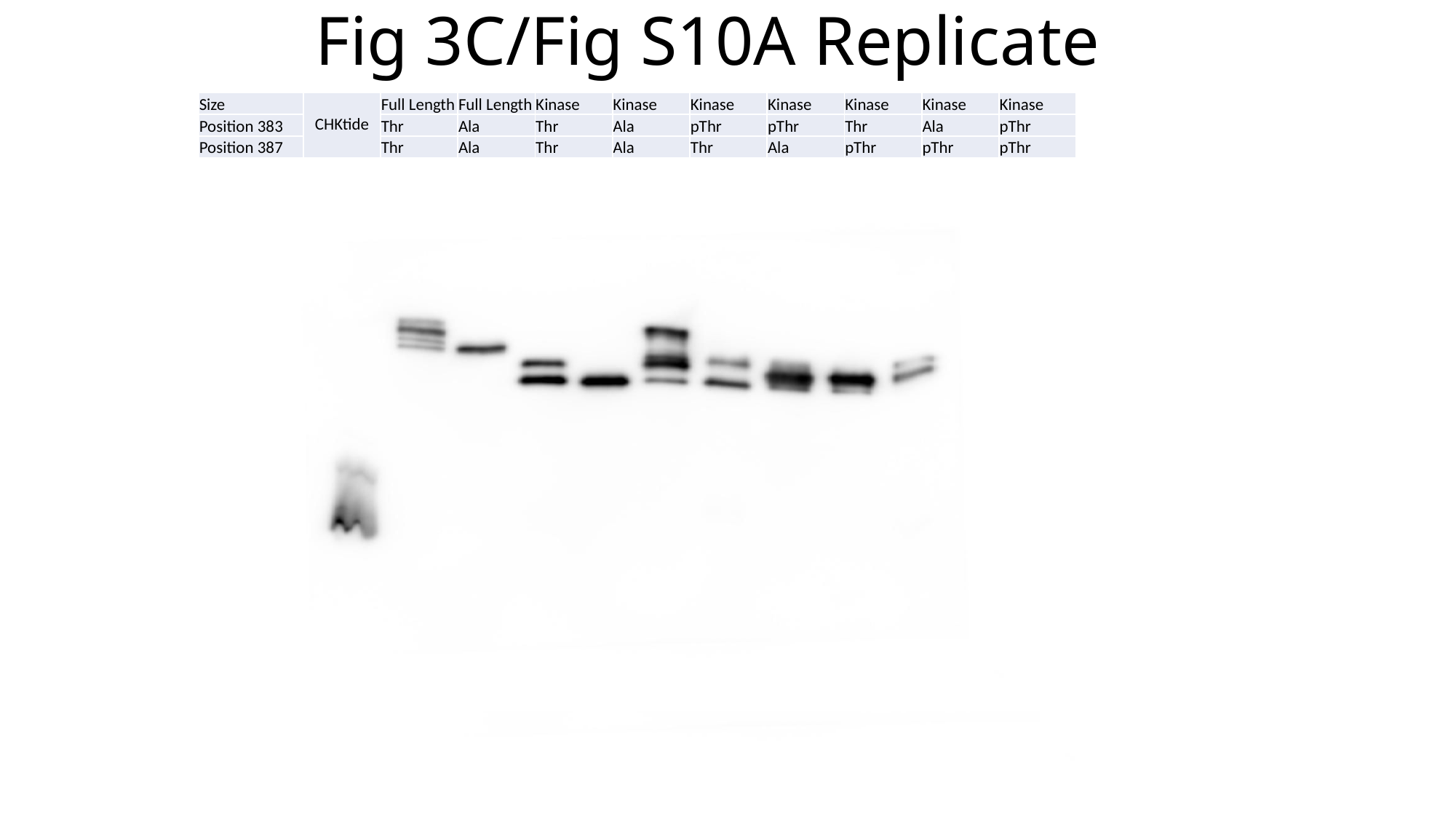

# Fig 3C/Fig S10A Replicate
| Size | CHKtide | Full Length | Full Length | Kinase | Kinase | Kinase | Kinase | Kinase | Kinase | Kinase |
| --- | --- | --- | --- | --- | --- | --- | --- | --- | --- | --- |
| Position 383 | | Thr | Ala | Thr | Ala | pThr | pThr | Thr | Ala | pThr |
| Position 387 | | Thr | Ala | Thr | Ala | Thr | Ala | pThr | pThr | pThr |

## Slide 5
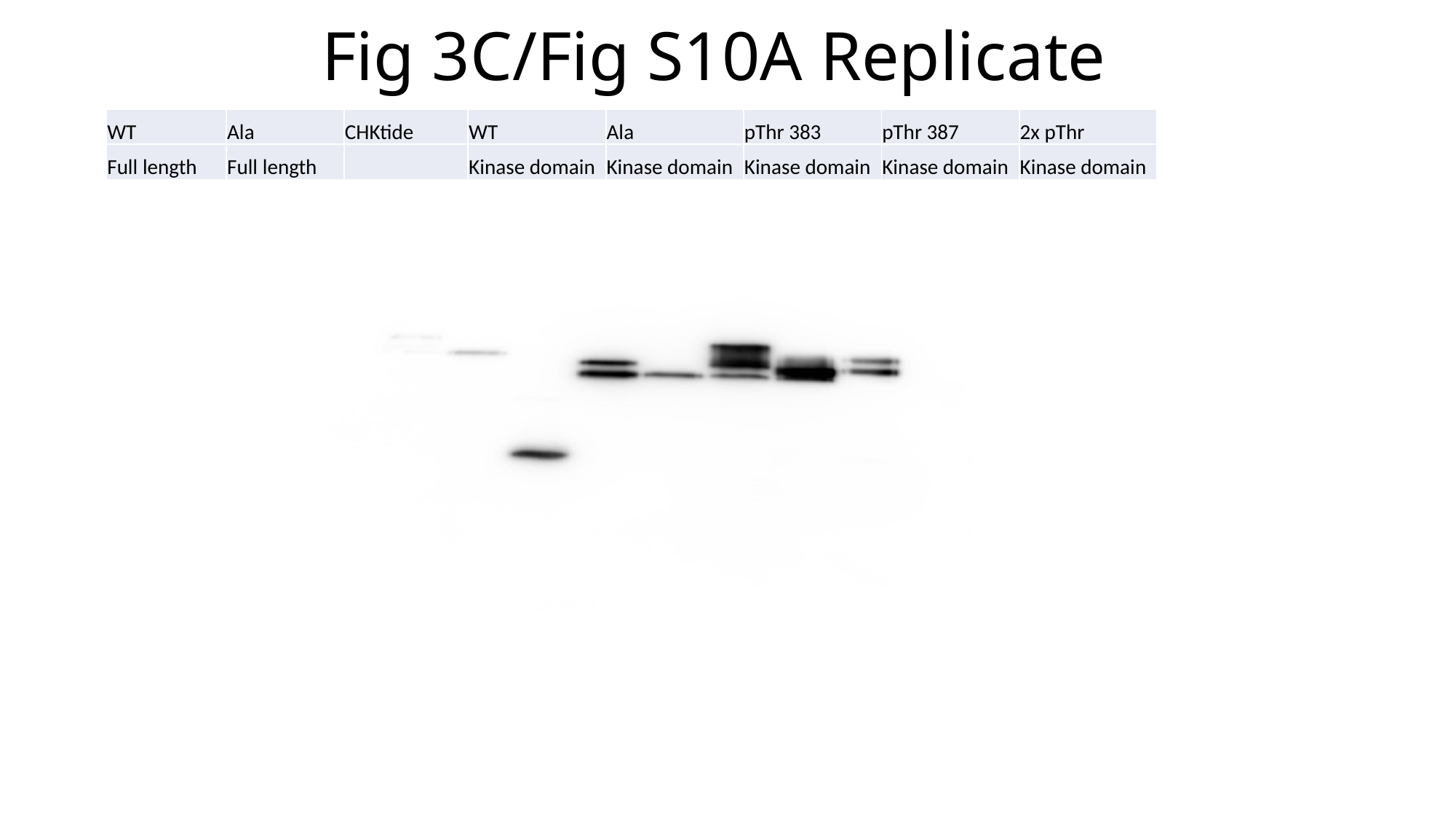

# Fig 3C/Fig S10A Replicate
| WT | Ala | CHKtide | WT | Ala | pThr 383 | pThr 387 | 2x pThr |
| --- | --- | --- | --- | --- | --- | --- | --- |
| Full length | Full length | | Kinase domain | Kinase domain | Kinase domain | Kinase domain | Kinase domain |

## Slide 6
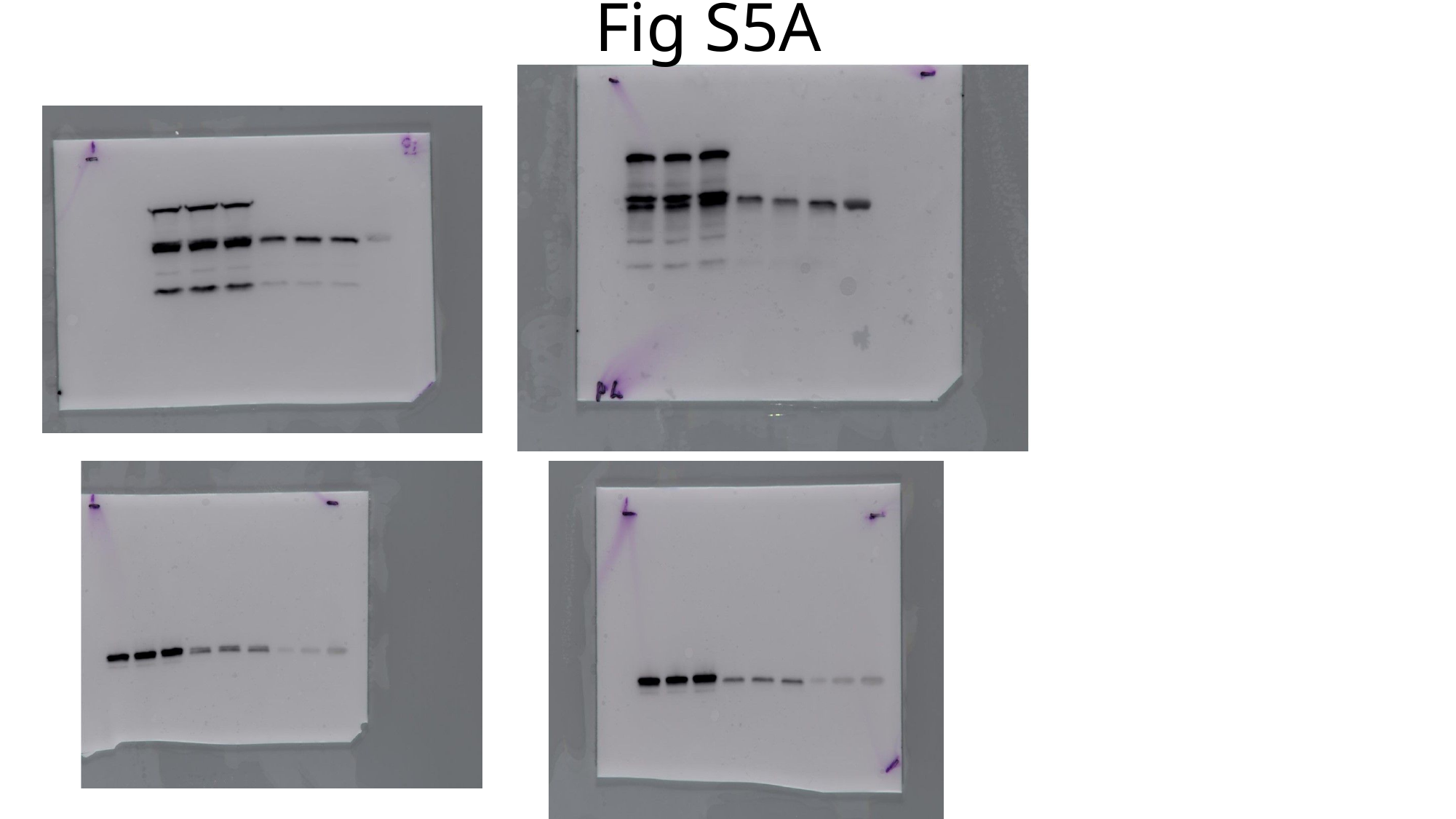

Fig S5A

## Slide 7
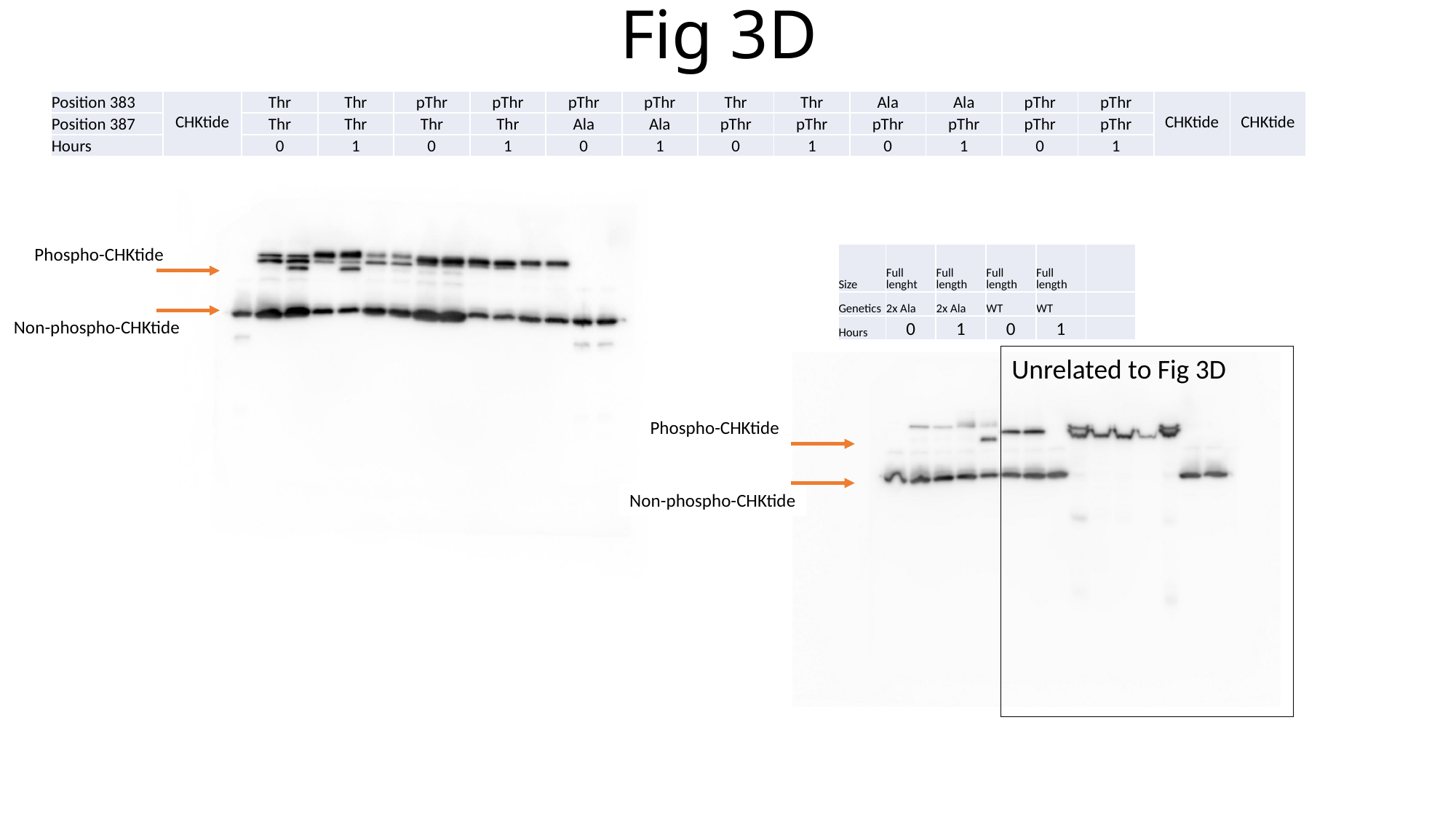

# Fig 3D
| Position 383 | CHKtide | Thr | Thr | pThr | pThr | pThr | pThr | Thr | Thr | Ala | Ala | pThr | pThr | CHKtide | CHKtide |
| --- | --- | --- | --- | --- | --- | --- | --- | --- | --- | --- | --- | --- | --- | --- | --- |
| Position 387 | | Thr | Thr | Thr | Thr | Ala | Ala | pThr | pThr | pThr | pThr | pThr | pThr | | |
| Hours | | 0 | 1 | 0 | 1 | 0 | 1 | 0 | 1 | 0 | 1 | 0 | 1 | | |
Phospho-CHKtide
| Size | Full lenght | Full length | Full length | Full length | |
| --- | --- | --- | --- | --- | --- |
| Genetics | 2x Ala | 2x Ala | WT | WT | |
| Hours | 0 | 1 | 0 | 1 | |
Non-phospho-CHKtide
Unrelated to Fig 3D
Phospho-CHKtide
Non-phospho-CHKtide

## Slide 8
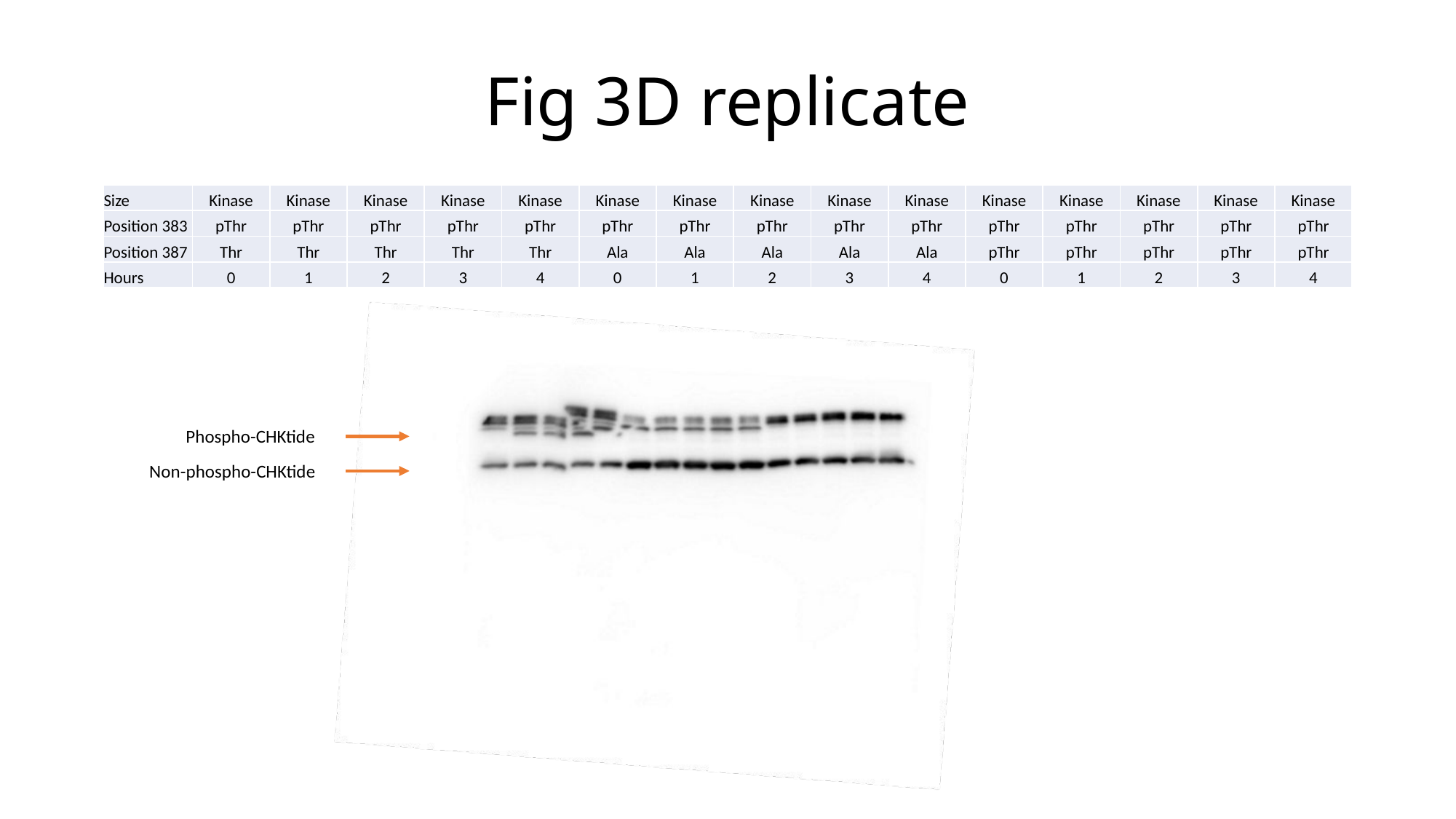

# Fig 3D replicate
| Size | Kinase | Kinase | Kinase | Kinase | Kinase | Kinase | Kinase | Kinase | Kinase | Kinase | Kinase | Kinase | Kinase | Kinase | Kinase |
| --- | --- | --- | --- | --- | --- | --- | --- | --- | --- | --- | --- | --- | --- | --- | --- |
| Position 383 | pThr | pThr | pThr | pThr | pThr | pThr | pThr | pThr | pThr | pThr | pThr | pThr | pThr | pThr | pThr |
| Position 387 | Thr | Thr | Thr | Thr | Thr | Ala | Ala | Ala | Ala | Ala | pThr | pThr | pThr | pThr | pThr |
| Hours | 0 | 1 | 2 | 3 | 4 | 0 | 1 | 2 | 3 | 4 | 0 | 1 | 2 | 3 | 4 |
Phospho-CHKtide
Non-phospho-CHKtide

## Slide 9
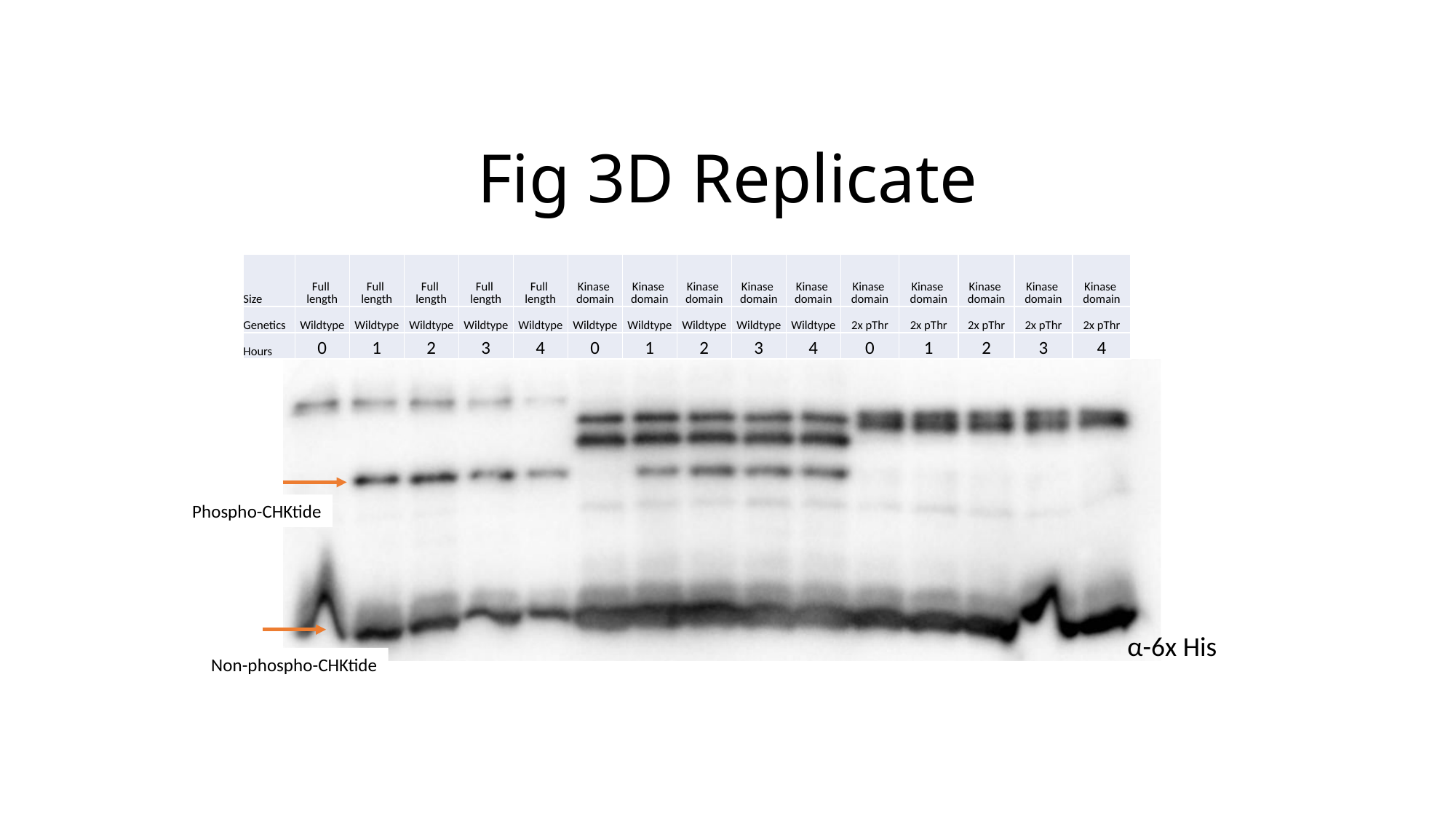

# Fig 3D Replicate
| Size | Full length | Full length | Full length | Full length | Full length | Kinase domain | Kinase domain | Kinase domain | Kinase domain | Kinase domain | Kinase domain | Kinase domain | Kinase domain | Kinase domain | Kinase domain |
| --- | --- | --- | --- | --- | --- | --- | --- | --- | --- | --- | --- | --- | --- | --- | --- |
| Genetics | Wildtype | Wildtype | Wildtype | Wildtype | Wildtype | Wildtype | Wildtype | Wildtype | Wildtype | Wildtype | 2x pThr | 2x pThr | 2x pThr | 2x pThr | 2x pThr |
| Hours | 0 | 1 | 2 | 3 | 4 | 0 | 1 | 2 | 3 | 4 | 0 | 1 | 2 | 3 | 4 |
Phospho-CHKtide
α-6x His
Non-phospho-CHKtide

## Slide 10
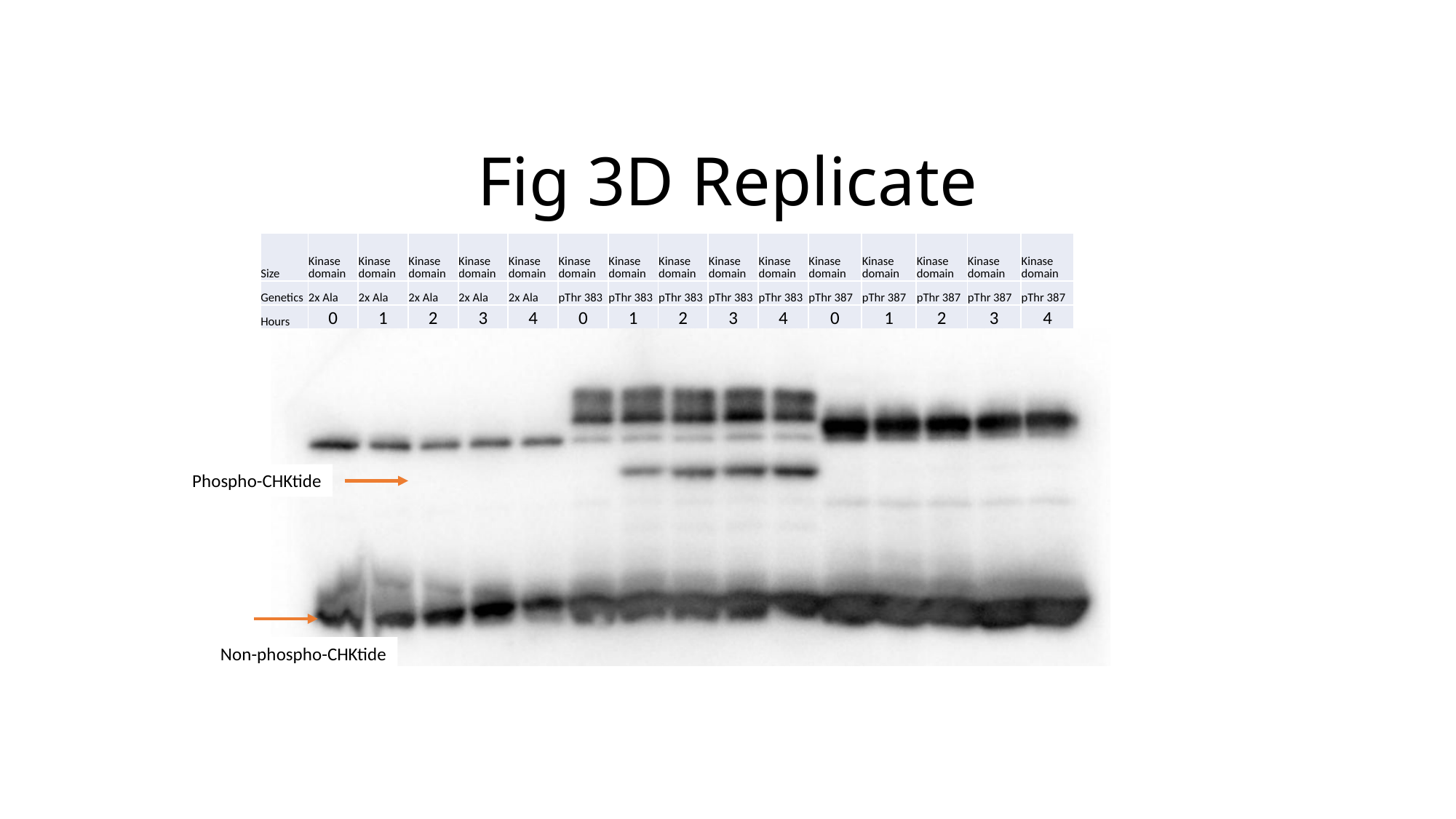

# Fig 3D Replicate
| Size | Kinase domain | Kinase domain | Kinase domain | Kinase domain | Kinase domain | Kinase domain | Kinase domain | Kinase domain | Kinase domain | Kinase domain | Kinase domain | Kinase domain | Kinase domain | Kinase domain | Kinase domain |
| --- | --- | --- | --- | --- | --- | --- | --- | --- | --- | --- | --- | --- | --- | --- | --- |
| Genetics | 2x Ala | 2x Ala | 2x Ala | 2x Ala | 2x Ala | pThr 383 | pThr 383 | pThr 383 | pThr 383 | pThr 383 | pThr 387 | pThr 387 | pThr 387 | pThr 387 | pThr 387 |
| Hours | 0 | 1 | 2 | 3 | 4 | 0 | 1 | 2 | 3 | 4 | 0 | 1 | 2 | 3 | 4 |
Phospho-CHKtide
Non-phospho-CHKtide

## Slide 11
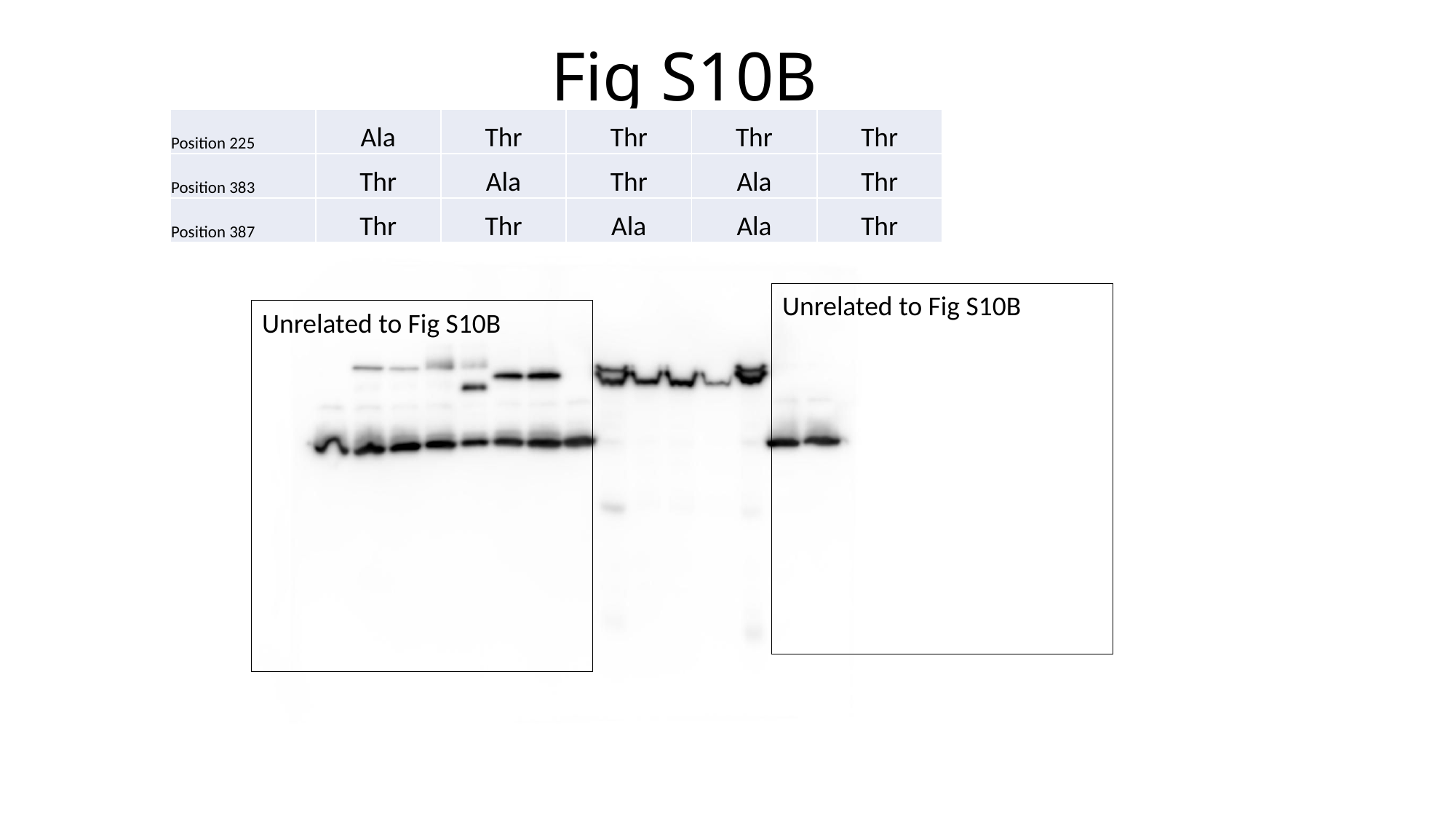

# Fig S10B
| Position 225 | Ala | Thr | Thr | Thr | Thr |
| --- | --- | --- | --- | --- | --- |
| Position 383 | Thr | Ala | Thr | Ala | Thr |
| Position 387 | Thr | Thr | Ala | Ala | Thr |
Unrelated to Fig S10B
Unrelated to Fig S10B

## Slide 12
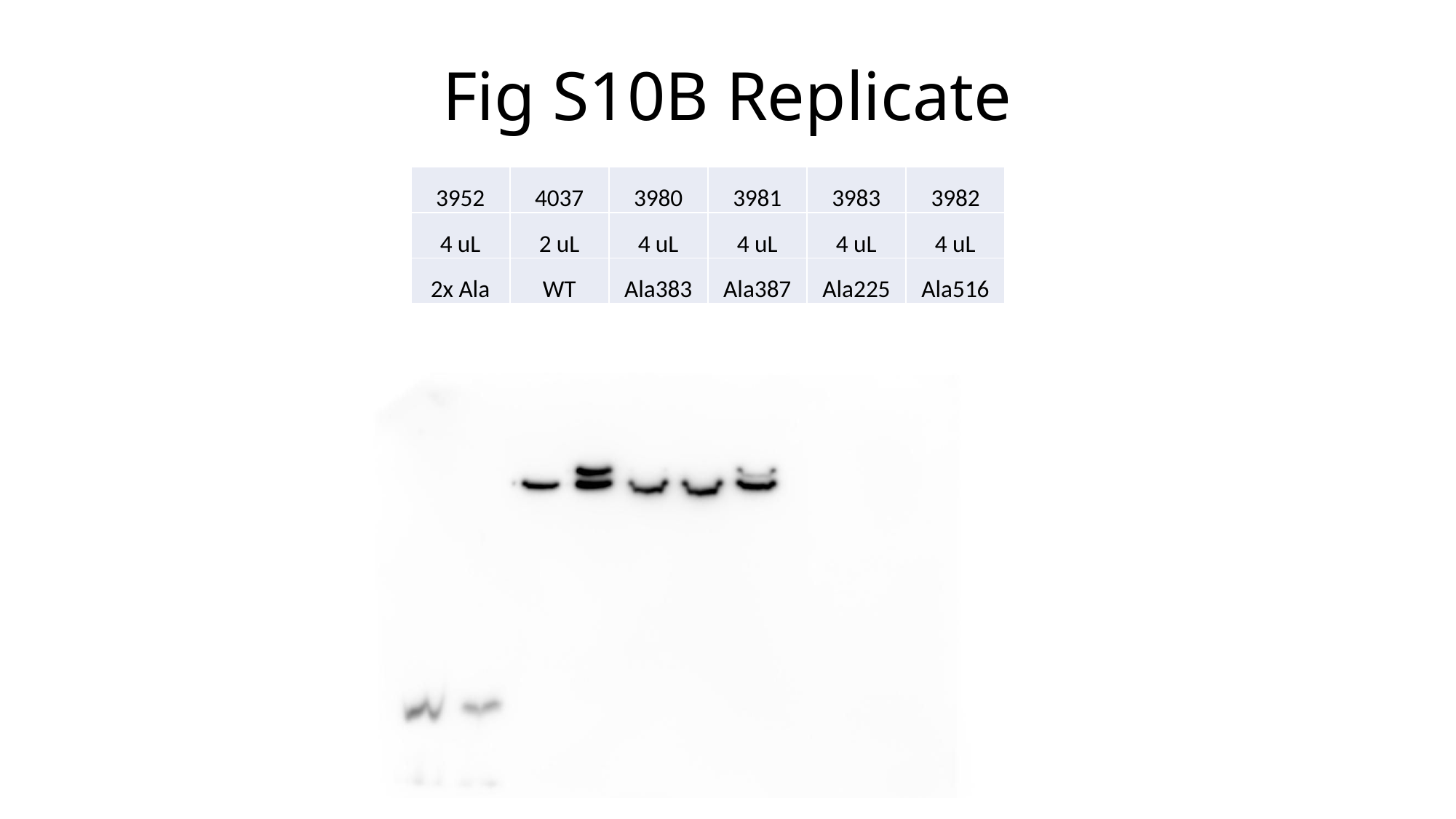

# Fig S10B Replicate
| 3952 | 4037 | 3980 | 3981 | 3983 | 3982 |
| --- | --- | --- | --- | --- | --- |
| 4 uL | 2 uL | 4 uL | 4 uL | 4 uL | 4 uL |
| 2x Ala | WT | Ala383 | Ala387 | Ala225 | Ala516 |
